# Interfacial Synergy in Hierarchical FMWCNTs/α-Fe_2_O_3_ and Ti_3_C_2_T_x_-MXene Electrodes for High-Performance Asymmetric Supercapacitors

**DOI:** 10.3390/polym18182262

**Published:** 2026-09-16

**Authors:** Abdul Mutlib, Jihyo Lim, Inkyum Kim, Aqsa Ghazal, Hyunwoo Cho, Ahsan Javed, Daewon Kim

**Affiliations:** 1 Department of Semiconductor Engineering, Kyung Hee University, 1732 Deogyeong-daero, Giheung-gu, Yongin 17104, Republic of Korea; abdul.mutlib@khu.ac.kr (A.M.); jihyo.lim@khu.ac.kr (J.L.); aqsa.ghazal@khu.ac.kr (A.G.); 2 Department of Electronic Engineering, Institute for Wearable Convergence Electronics, Kyung Hee University, 1732 Deogyeong-daero, Giheung-gu, Yongin 17104, Republic of Korea; inkyum.kim@khu.ac.kr; 3 Department of Electronics and Information Convergence Engineering, Kyung Hee University, 1732 Deogyeong-daero, Giheung-gu, Yongin 17104, Republic of Korea; hyunwoo.cho@khu.ac.kr; 4 Department of Physics, Syed Babar Ali School of Science and Engineering, Lahore University of Management Sciences (LUMS), Lahore 54792, Pakistan; ahsan.javed@lums.edu.pk

**Keywords:** asymmetric supercapacitors, energy storage, high energy density, functionalized MWCNT, α-Fe_2_O_3_, MXene

## Abstract

Asymmetric supercapacitors (ASCs) possess the potential to deliver high energy and power densities. However, current systems encounter limitations arising from complex charge-storage mechanisms and from interfacial interactions between electrolyte ions and electrode materials. Herein, the electrochemical response of functionalized multiwalled carbon nanotube (FMWCNT)/α-Fe_2_O_3_ electrodes in an alkaline KOH electrolyte is investigated. In contrast to pristine FMWCNTs and α-Fe_2_O_3_, the FMWCNTs/α-Fe_2_O_3_ heterostructure exhibits a higher specific surface area and porosity, enhancing electrolyte diffusion and access to active sites. A high specific capacitance of 1007.3 F/g is achieved at 4 A/g, attributed to an optimized nanostructure for OH^−^ induced Faradaic redox reactions. These experimental enhancements are consistent with first-principles density functional theory (DFT) calculations. In addition, an ASC utilizing the FMWCNTs/α-Fe_2_O_3_//Ti_3_C_2_T_x_-MXene configuration delivers a high energy density of 78.3 Wh/kg, a power density of 4072 W/kg, and excellent cycling stability, retaining 90% of the initial capacitance after 10,000 cycles. These findings establish the FMWCNTs/α-Fe_2_O_3_//Ti_3_C_2_T_x_-MXene configuration as a promising platform for advanced ASCs and provide mechanistic insights into the development of scalable and efficient energy storage devices.

## 1. Introduction

The current global environmental crisis underscores the urgent need for sustainable energy conversion and storage technologies [1]. The increasing reliance on renewable energy sources, including solar, wind, and ocean-generated energy, constitutes one of the main strategies proposed to address the issue and requires the deployment of highly effective and powerful energy storage systems. Consequently, the performance and reliability of storage devices remain highly critical to the successful implementation of these renewable resources. Supercapacitors (SCs) and lithium-ion batteries (LIBs) represent some of the most well-known energy storage technologies, gaining significant attention because of their applications in modern electronics and energy management [2]. SCs offer several advantages, including high power density, inherent safety, long cycle life, cost-effectiveness, fast charge–discharge rates, and environmental benignity. These features complement the limitations of conventional LIBs [3,4].

In high-power electronic applications, the design requirements of SCs aim to achieve high power density and simultaneously improve energy density and cycling stability [5,6,7,8]. These improvements are crucial for extending the operational lifespan of energy storage systems. Two major approaches have been pursued to achieve these goals: expanding the potential window and enhancing the specific capacitance of electrode materials based on the energy equation [9,10].

(1) $$E=\frac{1}{2}CV^{2}$$

These strategies have led to the development of various electrode enhancement methods, exemplified by the electrochemical synthesis of nanomaterials, the fabrication of nanocomposites, and the optimization of electrolytes in both organic and ionic systems. Recently, the emergence of hybrid energy storage systems combining different types of charge storage mechanisms has accelerated research in this field. In ASCs, the combination of two different electrode materials operating in an aqueous electrolyte allows charge storage via electric double-layer capacitance (EDLC) at one electrode and Faradaic pseudocapacitance reaction at the other, thereby exploiting the advantages of both mechanisms [11,12]. Asymmetric supercapacitors (ASCs) have been proposed as a promising strategy to enhance the energy-storage capability of conventional supercapacitors by integrating two different electrodes with complementary electrochemical properties. In a typical ASC, the positive electrode is generally composed of a Faradaic or pseudocapacitive material, whereas the negative electrode is usually based on highly conductive carbonaceous materials or other capacitive materials. The electrochemical compatibility, capacitance, potential window, and charge balance of the two electrodes strongly influence the overall performance of the assembled device. In particular, the rational coupling of a redox-active positive electrode with a highly conductive negative electrode can promote efficient charge transport, improve rate capability, and enhance cycling stability. In this work, the FMWCNTs/α-Fe_2_O_3_ composite is employed as the positive electrode, where the redox-active α-Fe_2_O_3_ component contributes Faradaic charge storage, while Ti_3_C_2_T_x_-MXene is employed as the negative electrode owing to its high electrical conductivity and favorable capacitive charge-storage characteristics. The complementary electrochemical characteristics of these two electrodes facilitate the construction of an ASC with improved overall electrochemical performance [13,14,15].

Beyond the selection of electroactive materials, polymeric components play an important role in the structural and electrochemical integration of quasi-solid-state supercapacitors. In particular, polymer binders can improve the mechanical cohesion and adhesion of electrode materials, helping to maintain intimate contact between the active components and current collector during repeated electrochemical cycling. In addition, gel polymer electrolytes provide a quasi-solid ion-conducting medium that can improve electrolyte retention and facilitate the construction of mechanically stable and compact energy-storage devices. Poly(vinylidene fluoride) (PVDF) is widely used as an electrode binder because of its chemical stability and strong binding capability, whereas poly(vinyl alcohol) (PVA)-based gel electrolytes provide a flexible polymeric matrix capable of retaining aqueous alkaline electrolytes. Therefore, the integration of polymeric components with electroactive nanostructures is an important consideration for developing stable and practical quasi-solid-state ASCs [16,17,18]. Transition metal oxides (TMOs)/hydroxides such as MnO_2_, WO_3_, V_2_O_5_, CuO, Fe_2_O_3_, and MoO_3_ have been the subject of considerable recent interest as electrode materials for SCs because they possess multiple redox states beneficial for electrochemical charge storage [19,20].

TMOs provide multiple redox states and high theoretical capacitance yet exhibit limited electrical conductivity and poor cycling stability, and these drawbacks require resolution for practical energy storage applications. To address these issues, TMOs have been coupled with conductive substrates such as multi-walled carbon nanotubes (MWCNTs). Carbon nanomaterials of this type, i.e., carbon nanotubes (CNTs), are regarded as efficient conductors due to their unique physical structure and robust sp^2^ covalent bonding between carbon atoms, and their exceptional mechanical strength enables the formation of effective conductive networks, rendering them highly promising candidates for high-performance electrode applications [21,22]. This coupling increases the conductivity and active surface area of the resulting composite and enhances electrochemical performance [23,24].

As an alternative to traditional carbon supports, electrochemical engineering of graphite architectures provides a strategy for tailoring the morphology and surface properties of carbon-based electrode materials. Electrochemical etching has been demonstrated to modify graphite surfaces and generate nanoscale structural features, thereby altering the accessible surface area and defect-related characteristics of the carbon framework. Such electrochemical treatment can expose additional electrochemically accessible sites and modify the interfacial properties of graphite, providing a controllable approach for tuning its electrochemical behavior [25]. Moreover, electrochemical studies have shown that the use of graphite as a conductive interface or current collector can influence electrode–electrolyte interactions, ionic transport, and the overall electrochemical performance of aqueous supercapacitor systems [26].

Iron oxide (Fe_2_O_3_) is a stable n-type semiconductor with a band gap of 2.0–2.2 eV and abundant redox-active sites, properties making it an appropriate electrode material for energy storage systems. However, its low intrinsic conductivity has prompted the need to utilize hybrid composites to achieve satisfactory performance [27,28]. Notably, Fe_2_O_3_ exhibits pseudocapacitive behavior during electrochemical cycling, thereby increasing the energy density of SCs via reversible Faradaic processes.

MXenes are a novel class of two-dimensional (2D) materials belonging to the family of transition metal carbides, nitrides, and carbonitrides with the general formula M_n+1_X_n_T_x_. Here, M represents an early transition metal, X denotes carbon or nitrogen, and T_x_ denotes surface terminations such as –OH, –O, or –F [29,30]. MXenes are hydrophilic and possess outstanding electrical conductivity, a large surface area, high thermal stability, and solution processability [31,32]. Consequently, Ti_3_C_2_T_x_-MXene has been employed as an anode material in ASCs to improve the energy density, cycling stability, and overall electrochemical performance of the resulting devices.

Although significant developments have occurred in Fe_2_O_3_/carbon-based SC electrodes, achieving simultaneous improvement in electronic conductivity, ion-accessible porosity, and interfacial charge-transfer kinetics remains a significant challenge due to Fe_2_O_3_ agglomeration and sluggish OH^−^ diffusion. The combined role of defect-related oxygen species and interfacial electronic coupling between conductive carbon frameworks and α-Fe_2_O_3_ nanospheres (NSs) remains poorly explained and has only recently begun to be examined using first-principles calculations [32]. Therefore, engineering a hierarchical FMWCNTs/α-Fe_2_O_3_ HTs capable of promoting fast electron transport, efficient OH^−^ adsorption/desorption kinetics, and stable Faradaic reactions is highly desirable for next-generation ASCs.

In this work, a defect-rich hierarchical FMWCNTs/α-Fe_2_O_3_ heterostructure is engineered through conductive interfacial coupling between functionalized multi-walled carbon nanotubes and α-Fe_2_O_3_ NSs. The interconnected FMWCNT network not only suppresses α-Fe_2_O_3_ agglomeration but also promotes interfacial electronic interaction, thereby improving the kinetics of diffusion-controlled Faradaic charge storage. The hybrid HTs exhibited improved electrochemical performance compared to pristine FMWCNTs and α-Fe_2_O_3_ due to the hierarchical porous structure, which enhanced electrolyte accessibility and the utilization of active sites. DFT calculations indicate enhanced electronic conductivity and accelerated interfacial charge transfer kinetics. Furthermore, integrating FMWCNTs/α-Fe_2_O_3_ with Ti_3_C_2_T_x_-MXene in an asymmetric device delivered a high energy density (78.3 Wh/kg), a high power density (4072 W/kg), and excellent cycling durability. Practical viability was demonstrated by powering an LED, confirming the potential of this system for next-generation high-performance energy storage devices. Overall, this work demonstrates the integration of FMWCNTs/α-Fe_2_O_3_ HTs with Ti_3_C_2_T_x_-MXene as an effective strategy for developing advanced ASCs with high energy and power densities.

## 2. Materials and Methods

### 2.1. Materials

Reagents used in this study were of analytical grade and utilized without further purification. Iron(III) nitrate nonahydrate (Fe(NO_3_)_3_·9H_2_O, 99.99%) and ethylenediamine (C_2_H_8_N_2_, 99.99%) were used for the synthesis of α-Fe_2_O_3_ NSs. Potassium hydroxide (KOH, 99.99%), poly(vinylidene difluoride) (PVDF, 99.99%), and N-methyl-2-pyrrolidone (NMP, 99%) were employed as the electrolyte, binder, and slurry solvent, respectively. All of these reagents were purchased from Sigma-Aldrich, Inc. (St. Louis, MO, USA). Multi-walled carbon nanotubes (MWCNTs) with a diameter of 50–90 nm and >95% carbon purity were also purchased from Sigma-Aldrich, Inc. (St. Louis, MO, USA). Activated carbon (AC) and nickel foam (NF) substrates were purchased from MTI Korea (Seoul, South Korea). All experiments were performed using deionized (DI) water with a resistivity of 18 MΩ·cm.

### 2.2. Synthesis of Ti_3_C_2_T_x_-MXene

Ti_3_AlC_2_ powder (purity > 98 wt%) was obtained from Forsman Co., Ltd. (Beijing, China). For the etching process, 2 g of Ti_3_AlC_2_ powder was dispersed in 20 mL of hydrofluoric acid (HF, >40 wt%) and stirred at 60 °C for 24 h to remove the Al layer. The mixture was then centrifuged and repeatedly washed with DI water until the pH reached 6, indicating the removal of HF residues. Finally, the Ti_3_C_2_T_x_-MXene powder was obtained by vacuum drying the powder at 80 °C for 24 h [33].

### 2.3. Synthesis of α-Fe_2_O_3_

A hydrothermal method was used to produce the α-Fe_2_O_3_ NSs. Specifically, 1.2 g of Fe(NO_3_)_3_·9H_2_O was dissolved in 150 mL of DI water under vigorous stirring. Subsequently, under continuous stirring, ethylenediamine (11 mL) was added dropwise until the pH reached approximately 11. The suspension was mixed for 30 min to ensure homogeneity and then transferred into a Teflon-lined stainless-steel autoclave. The autoclave was sealed, heated to 150 °C, and maintained at this temperature for 12 h. The resulting product was centrifuged and washed several times with ethanol and DI water, and dried at 60 °C for 2 h. Finally, a calcination step was carried out at 500 °C for 2 h at a heating rate of 10 °C/min to obtain α-Fe_2_O_3_ NSs [34].

### 2.4. Functionalization of MWCNTs

MWCNTs were functionalized on their surfaces via an acid treatment assisted by ultrasound. Specifically, 0.5 g of MWCNTs was dispersed into 100 mL of a mixed acid solution composed of concentrated nitric acid (HNO_3_) and sulfuric acid (H_2_SO_4_) in a volume ratio of 1:3. The suspension was sonicated in a water bath at 40 °C for 10 h to introduce oxygen-containing functional groups, mainly carboxyl (–COOH) groups, onto the nanotube surfaces. Following sonication, the product was filtered and thoroughly washed with DI water until a neutral pH (pH 7) was reached. The resulting functionalized MWCNTs were dried at 40 °C for 10 h and stored for subsequent use [35].

### 2.5. Synthesis of FMWCNTs/α-Fe_2_O_3_ Composite Heterostructures

**Scheme 1:**
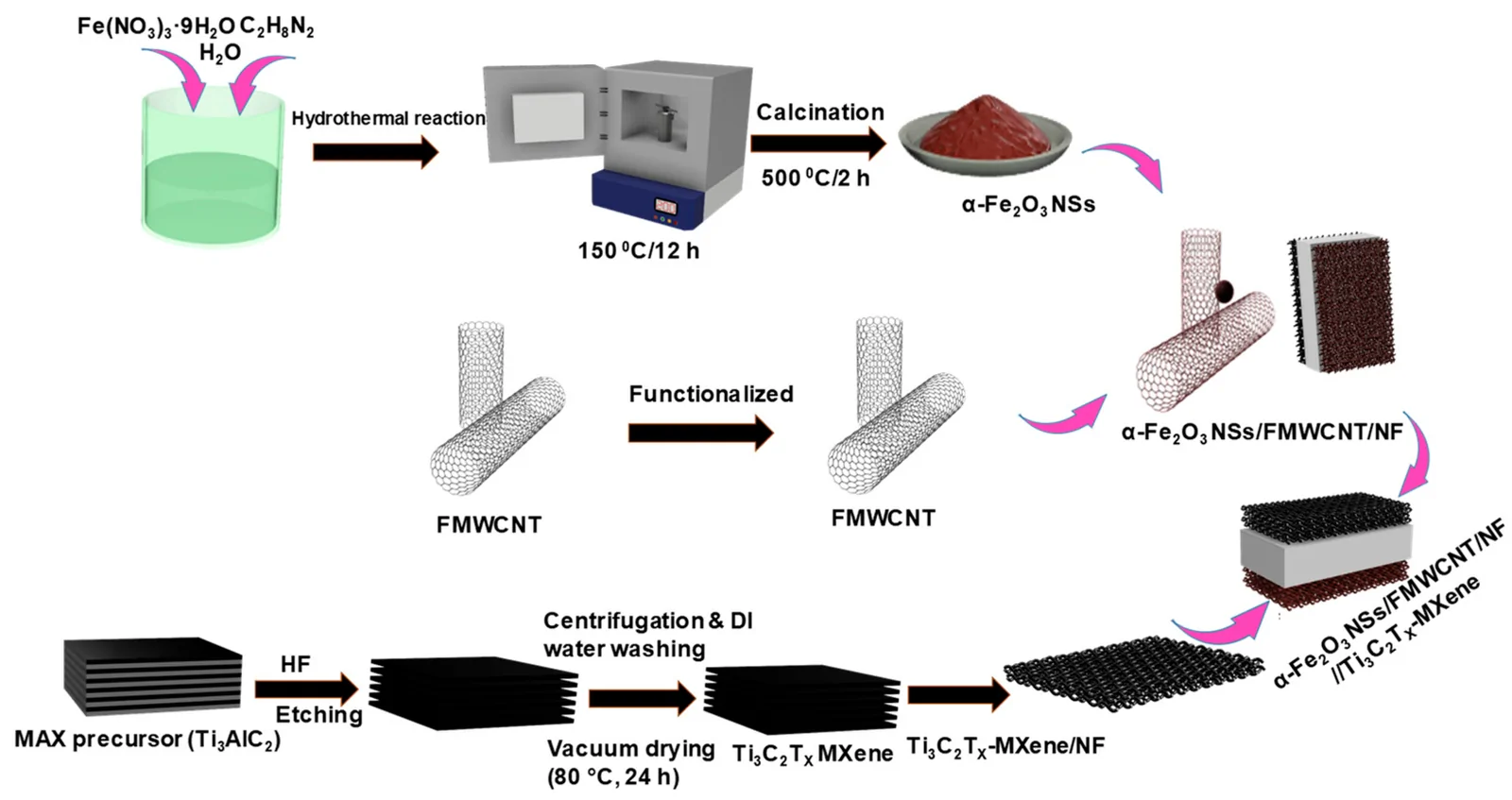
Schematic illustration of the synthesis of α-Fe_2_O_3_ NSs, FMWCNTs, and Ti_3_C_2_T_x_-MXene, the fabrication of the FMWCNTs/α-Fe_2_O_3_ composite, and the final assembly of the ASC device.

The FMWCNTs/α-Fe_2_O_3_ composite was prepared by dispersing 0.4 g of FMWCNTs and 0.4 g of α-Fe_2_O_3_ in 80 mL of DI water, and the dispersion was continuously stirred at 500 rpm for 30 min. The mixture was then sealed in a Teflon-lined stainless steel autoclave and heated at 200 °C for 24 h to promote composite formation. After naturally cooling to room temperature, the product was removed, filtered, and repeatedly washed with DI water to eliminate residual impurities and unreacted species. The final composite was dried at 70 °C for 8 h to remove residual moisture. The overall synthesis procedures of the α-Fe_2_O_3_ NSs, FMWCNTs, and Ti_3_C_2_T_x_-MXene, together with the fabrication process of the ASC, are schematically illustrated in Scheme 1.

### 2.6. Computational Details

First-principles calculations based on density functional theory (DFT) were performed using the Vienna Ab initio Simulation Package (VASP; VASP Software GmbH, Vienna, Austria). The generalized gradient approximation (GGA) with the Perdew–Burke–Ernzerhof (PBE) functional was used to describe the exchange–correlation interactions. The projector augmented-wave (PAW) method was employed with a plane-wave cutoff energy of 500 eV. To account for the correlation effects of the Fe 3d electrons in α-Fe_2_O_3_, the DFT+U method was applied using an effective Hubbard parameter (*U*_eff_) of 4.3 eV. The Brillouin zone was sampled using a 3 × 3 × 1 Monkhorst–Pack k-point mesh. The atomic positions were fully relaxed until the total energy and ionic forces converged to 10^−5^ eV and 0.02 eV Å^−1^, respectively.

### 2.7. Characterization Tools

X-ray diffraction (XRD, M18XHF-SRA, MAC Science Co., Ltd., Yokohama, Japan; radiation source: Cu Kα, λ = 1.5406 Å) analysis was performed to investigate the crystallographic structure and composition of the synthesized materials: FMWCNTs, α-Fe_2_O_3_, Ti_3_C_2_T_x_-MXene, and FMWCNTs/α-Fe_2_O_3_ electrodes. Field-emission scanning electron microscopy (FE-SEM, LEO SUPRA 55, Carl Zeiss, Oberkochen, Germany) and energy-dispersive X-ray spectroscopy (EDS) were used to examine the surface morphology and the distribution of elements. High-resolution transmission electron microscopy (HR-TEM) was conducted on a JEM-2100F microscope (JEOL Ltd., Akishima, Tokyo, Japan) equipped with an Oxford Instruments EDS detector (Oxford Instruments NanoAnalysis, High Wycombe, UK) for nanoscale structural characterization and elemental mapping. To determine chemical stoichiometry and electronic state, X-ray photoelectron spectroscopy (XPS, Multilab 2000, Thermo Electron, East Grinstead, UK) was used to analyze the samples. The surface area and porosity were measured from N_2_ adsorption–desorption isotherms using Brunauer–Emmett–Teller (BET) and Barrett–Joyner–Halenda (BJH) analyses on a BELSORP MAX (MP) analyzer (BEL Japan Inc., Osaka, Japan).

### 2.8. Electrochemical Measurements (Three-Electrode System)

The working electrodes were prepared by mixing the active material (FMWCNTs, α-Fe_2_O_3_, FMWCNTs/α-Fe_2_O_3_, or Ti_3_C_2_T_x_-MXene), AC, and PVDF binder in a mass ratio of 8:1:1 using NMP as the solvent. The mixture was sonicated for 20 min to form a uniform slurry, which was then coated onto nickel foam (1 cm × 2 cm), serving as the current collector. The coated electrodes were dried at 60 °C for 12 h to remove residual solvents and ensure good adhesion. PVDF was used as a polymeric binder to provide mechanical cohesion and promote adhesion of the active materials onto the nickel-foam current collector. The polymer binder helps maintain intimate contact among the FMWCNTs/α-Fe_2_O_3_ or Ti_3_C_2_T_x_-MXene particles and the current collector during repeated electrochemical cycling. For all synthesized samples, the mass loading of the active material deposited on the NF was maintained at 2 mg (1.0 mg cm^−2^), whereas that of the bare Ti_3_C_2_T_x_-MXene electrode was 3.5 mg (1.75 mg cm^−2^), considering the coated geometric area of 1 × 2 cm^2^. The controlled areal loading of ~1 mg cm^−2^ was chosen to minimize mass transport limitations while providing sufficient active material for reliable electrochemical evaluation, in accordance with the charge-balance criterion described in Section 3.8. Electrochemical performance was evaluated in a three-electrode configuration using 3 M KOH aqueous electrolyte. The prepared materials served as the working electrode, while a platinum wire and an Ag/AgCl (saturated KCl) electrode served as the counter and reference electrodes, respectively.

Cyclic voltammetry (CV), galvanostatic charge/discharge (GCD), and electrochemical impedance spectroscopy (EIS) were performed to analyze the charge-storage behavior and electrochemical kinetics of the synthesized electrode materials. These measurements were performed using an IviumStat electrochemical workstation (Ivium Technologies B.V., Eindhoven, The Netherlands). Standard equations were used to calculate specific capacitance, specific capacity, energy density, and power density as follows [36,37].

(2) $$C_{s}{\;[F\;g}^{-1}]=\frac{1}{mv\left(V_{f}-V_{i}\right)}\int_{Vi}^{Vf}I(V)dV$$

(3) $$Q_{s}{\;[C\;g}^{-1}]=\frac{1}{mv}\int_{Vi}^{Vf}I(V)dV$$

where *I* indicates the current, *v* denotes the scan rate, *m* is the mass of the active material, *t* denotes the discharge time, and (*V*_f_ − *V*_i_) corresponds to the applied potential window. GCD measurements were also conducted for each electrode, and the values of *C*_s_ and *Q*_s_ were calculated using Equations (4) and (5), respectively.

(4) $$C_{s}\;[{F\;g}^{-1}]=\frac{It}{m\left(V_{f}-V_{i}\right)}$$

(5) $$Q_{s}{\;[C\;g}^{-1}]=\frac{It}{m}$$

The energy density (*ED*) and power density (*PD*) of the fabricated device were calculated from these profiles using Equations (6) and (7), respectively.

(6) $$ED\;[{\mathrm{Wh}\;\mathrm{kg}}^{-1}]=\frac{0.5\times C_{sp}\times V^{2}}{3.6}$$

(7) $$PD{\;[W\;\mathrm{kg}}^{-1}]=\frac{3600\times E}{t_{d}}$$

where *C*_sp_ represents the specific capacitance of the device, *V* denotes the operating voltage window, and *t*_d_ is the discharge time.

### 2.9. Device (Two-Electrode) Fabrication

The electrodes for the two-electrode device were prepared following the same slurry coating procedure described in Section 2.8, using nickel foam (1 cm × 2 cm) as the current collector, and were dried at 60 °C to remove residual solvent. The SC device was assembled using FMWCNTs/α-Fe_2_O_3_ as the cathode and Ti_3_C_2_T_x_-MXene as the anode. A gel electrolyte was prepared by dissolving 3 g of polyvinyl alcohol (PVA) in 30 mL of DI water under continuous stirring at 70 °C. Once fully dissolved, 2.7 mL of 3 M KOH solution was added dropwise, and the mixture was stirred for an additional 2 h to form a homogeneous gel electrolyte. The PVA matrix serves as a polymeric host for the KOH electrolyte, providing a quasi-solid ion-conducting medium between the positive and negative electrodes. The polymer matrix also improves electrolyte retention and contributes to the mechanical stability of the assembled device [38]. Before assembly, the electrodes were soaked in the gel electrolyte to ensure proper infiltration and were subsequently dried at room temperature. The device was then assembled in a face-to-face configuration with a cellulose paper separator placed between the electrodes, and the assembly was sealed with Teflon tape, leaving a small portion of each electrode exposed for electrical contact. To maintain charge balance between the cathode and anode electrodes, the mass ratio of the two electrodes was adjusted based on the principle of charge balance using Equation (8):

(8) $$\frac{m_{+}}{m_{-}}=\frac{C_{sp-}\times \Delta V_{-}}{C_{sp+}\times \Delta V_{+}}$$

In Equation (8), m denotes the mass of the active material, *C*_sp+_ and *C*_sp−_ are the specific capacitances (F g^−1^) of the cathode and anode, and Δ*V*_+_ and Δ*V*_−_ represent their respective potential windows (*V*).

## 3. Results

### 3.1. Structural Studies

**Figure 1:**
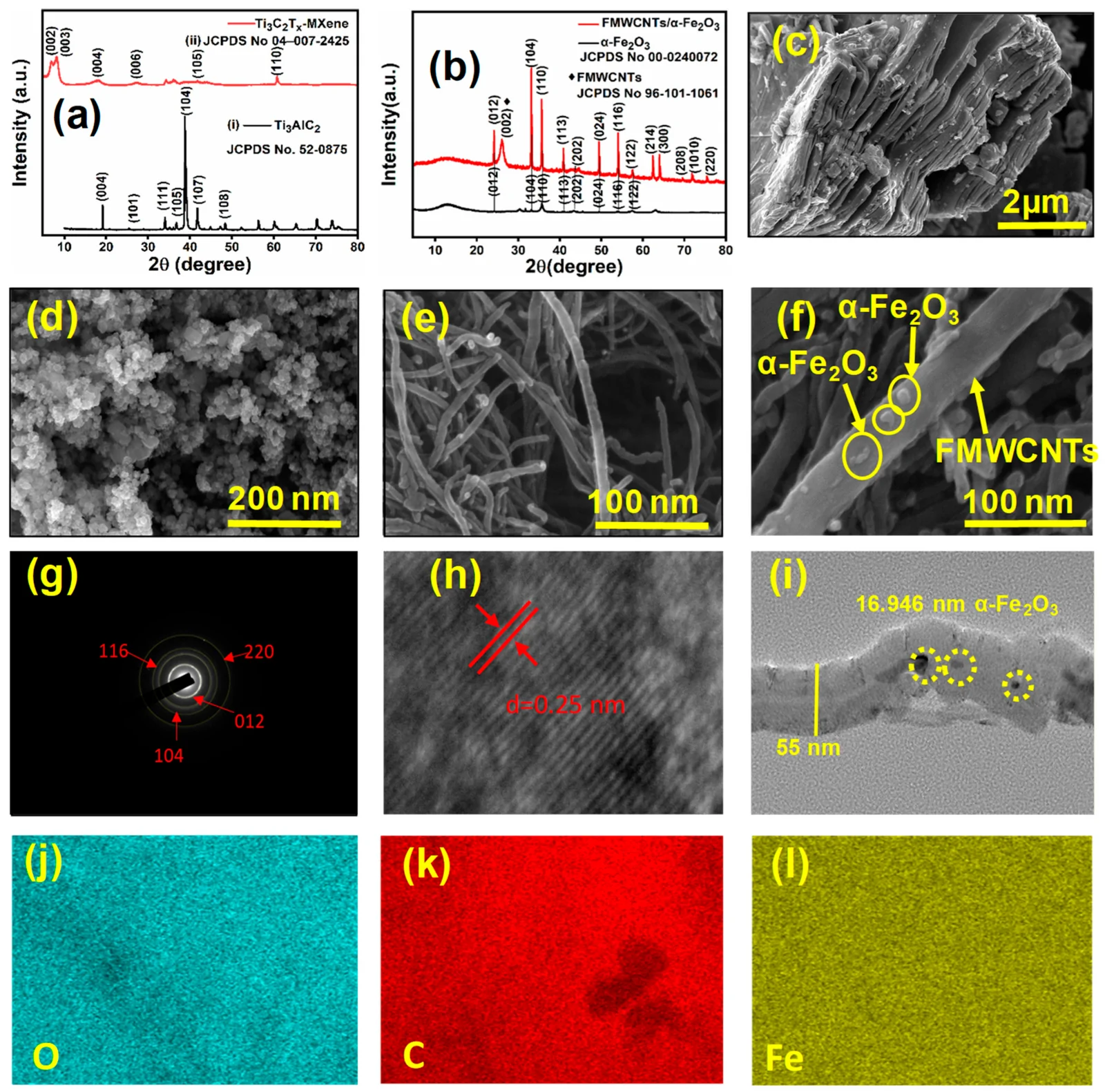
Structural and morphological characterization of the synthesized materials. (**a**) XRD patterns of (i) Ti_3_AlC_2_ MAX phase and (ii) Ti_3_C_2_T_x_-MXene. (**b**) XRD patterns of α-Fe_2_O_3_ and FMWCNTs/α-Fe_2_O_3_ composite, where the symbol ‘◆’ represents the peaks of FMWCNTs. (**c**–**f**) FE-SEM images of (**c**) Ti_3_C_2_T_x_-MXene nanosheets, (**d**) α-Fe_2_O_3_ NSs, (**e**) FMWCNTs, and (**f**) FMWCNTs/α-Fe_2_O_3_ hierarchical HTs. (**g**) SAED pattern showing diffraction rings, (**h**) HR-TEM image displaying lattice fringes, and (**i**) Low-magnification TEM image of FMWCNTs/α-Fe_2_O_3_ showing α-Fe_2_O_3_ NSs (~16.9 nm in diameter, *n* = 3) anchored on a nanotube (~55 nm in diameter, *n* = 1), with diameter measurements obtained using ImageJ (version 1.54). (**j**–**l**) EDS elemental mapping images corresponding to (**j**) O, (**k**) C, and (**l**) Fe.

XRD analysis was performed to study the crystallographic properties of the synthesized materials, and the results are presented in Figure 1a,b. The phase evolution and structural transformation were verified by analyzing the XRD patterns across all synthesis stages, including Ti_3_AlC_2_ MAX phase, Ti_3_C_2_T_x_-MXene, α-Fe_2_O_3_, and FMWCNTs/α-Fe_2_O_3_ composites. The diffraction peaks of Ti_3_AlC_2_ (Figure 1a(i)) are consistent with the standard card (JCPDS No. 52-0875), with characteristic reflections at ~19° and ~39° corresponding to the (004) and (104) planes, respectively [39]. After selective etching of the Al layer, a clear structural modification is detected in Figure 1a(ii) with the disappearance of the (104) peak at ~39°, confirming the removal of Al atomic layers from the MAX phase. This change is accompanied by a prominent shift of the (002) peak to lower 2*θ* values and an increase in its intensity, a typical signature of Ti_3_C_2_T_x_-MXene formation caused by interlayer-spacing expansion and exfoliation of the layered structure. The additional diffraction peaks adjacent to the (002) reflection do not indicate an independent (003) reflection. Instead, multiple peaks in this region reflect heterogeneous interlayer spacings across the multilayered Ti_3_C_2_T_x_ MXene. These variations in interlayer distance could be associated with the intercalation or retention of water molecules and/or residual ions between the MXene layers following the etching and washing processes [40,41]. Thus, the disappearance of the (104) reflection and the low-angle shift of the (002) peak together confirm the successful conversion from Ti_3_AlC_2_ to Ti_3_C_2_T_x_-MXene [42,43].

The XRD pattern for α-Fe_2_O_3_ NSs (Figure 1b) exhibits prominent diffraction peaks at 2θ values of 23.9°, 33.04°, 35.7°, 40.9°, 43.3°, 49.6°, 54.0°, and 57.5°. These peaks are indexed to the (012), (104), (110), (113), (202), (024), (116), and (122) planes of rhombohedral hematite α-Fe_2_O_3_ (JCPDS No. 00-024-0072). For the FMWCNTs/α-Fe_2_O_3_ composite, characteristic peaks of FMWCNTs at 26.2° (JCPDS No. 96-101-1061) and α-Fe_2_O_3_ (24.1°, 33.09°, 35.6°, 40.7°, 43.5°, 49.3°, 54.5°, and 57.5°; JCPDS No. 00-024-0072) are observed. Additional peaks at 2*θ* values of 62.5°, 64°, 69.4°, 71.6°, and 75.5° are also observed after composite formation, further confirming the rhombohedral structure of α-Fe_2_O_3_. The composite formation is further supported by the reduced intensity and slight broadening of the α-Fe_2_O_3_ reflections, attributed to the nanoscale grain size and intimate contact with the FMWCNT framework [44,45].

### 3.2. Surface Morphological Studies

FE-SEM was employed to characterize the surface morphology of the prepared materials. Figure 1c–f presents the surface structures of Ti_3_C_2_T_x_-MXene, pristine α-Fe_2_O_3_, FMWCNTs, and the FMWCNTs/α-Fe_2_O_3_ composite, respectively. As shown in Figure 1c, the Ti_3_C_2_T_x_-MXene exhibits a characteristic multi-layered, accordion-like morphology with localized exfoliated regions. This layered architecture confirms the successful selective removal of Al atomic layers from the MAX phase, effectively widening the interlayer spacing and providing well-defined channels that facilitate rapid intercalation and deintercalation of electrolyte ions. Although delaminated single-/few-layer Ti_3_C_2_T_x_ generally provides a higher accessible surface area and improved electrolyte-ion accessibility, multilayer Ti_3_C_2_T_x_ was deliberately selected because it retains the layered framework without requiring additional intercalation and delamination steps. This approach simplifies the processing procedure while maintaining the structural integrity of the MXene during electrode fabrication. Thus, the multilayer architecture provides a practical balance between ion accessibility, structural integrity, and ease of electrode fabrication [46,47,48,49]. Pristine α-Fe_2_O_3_ (Figure 1d) exhibits a nanospherical morphology, whereas the FMWCNTs (Figure 1e) present an entangled nanotube microstructure. In the FMWCNTs/α-Fe_2_O_3_ composite (Figure 1f), the α-Fe_2_O_3_ NSs are clearly observed to be distributed and anchored on the FMWCNT network, confirming the formation of the heterostructure. The interconnected FMWCNT framework decorated with α-Fe_2_O_3_ NSs, which contain octahedrally coordinated Fe–O units, forms a hierarchical architecture that can provide accessible pathways for electrolyte-ion transport and facilitate efficient ion diffusion during charge–discharge processes. The distribution of nanotubes and NSs in the composite increases the surface area and the number of electrochemically active sites, thus enhancing pseudocapacitive performance [50]. This hierarchical structure may promote efficient ion diffusion and mitigate structural stress during long-term cycling.

Figure 1g presents the SAED pattern of the FMWCNTs/α-Fe_2_O_3_ composite, in which the clear diffraction rings are indexed to the (012), (104), (116), and (220) planes of α-Fe_2_O_3_. The well-defined diffraction rings indicate the polycrystalline nature and good crystallinity of α-Fe_2_O_3_ NSs. The HR-TEM images provide detailed information on the crystalline nature of the α-Fe_2_O_3_ embedded in the composite matrix. The HR-TEM image (Figure 1h) displays clear lattice fringes with a d-spacing of 0.25 nm, corresponding to the (110) plane of α-Fe_2_O_3_. The SEM (Figure 1f) and low-magnification TEM (Figure 1i) images confirm the homogeneous dispersion of α-Fe_2_O_3_ NSs along the surfaces of the FMWCNTs. The α-Fe_2_O_3_ NSs exhibit an average diameter of approximately 16.9 nm, and the FMWCNTs show an average diameter of around 55 nm (Figure 1i), uniformly decorated by the α-Fe_2_O_3_ NSs. Such a homogeneous distribution results from the abundant nucleation sites provided by the oxygen-containing groups on the FMWCNT surfaces, onto which iron oxide species redeposit through a dissolution–precipitation mechanism during the hydrothermal treatment.

Elemental composition was determined by energy-dispersive X-ray spectroscopy (EDS). Elemental mapping images (Figure 1j–l) of FMWCNTs/α-Fe_2_O_3_ confirm the uniform spatial distribution of O, C, and Fe throughout the composite. The atomic percentages of O (18.82%), C (75.58%), and Fe (5.6%), obtained from the EDS spectrum and corresponding quantitative analysis presented in Figure S1, confirm the incorporation of α-Fe_2_O_3_ onto the FMWCNTs without significant agglomeration. The relatively low Fe content is attributed to the strong carbon background from the volumetrically dominant MWCNT matrix within the electron-beam interaction volume and to the nanoscale dispersion of α-Fe_2_O_3_, and it therefore reflects the locally scanned surface region rather than the overall bulk composition. This homogeneous distribution is considered critical for efficient charge transfer and improved electrochemical performance. The hierarchical architecture with integrated nanoscale features is expected to maximize the electrochemical surface area and facilitate rapid ion transport crucial for high-performance SC operation.

### 3.3. Elemental and Compositional Analysis

**Figure 2:**
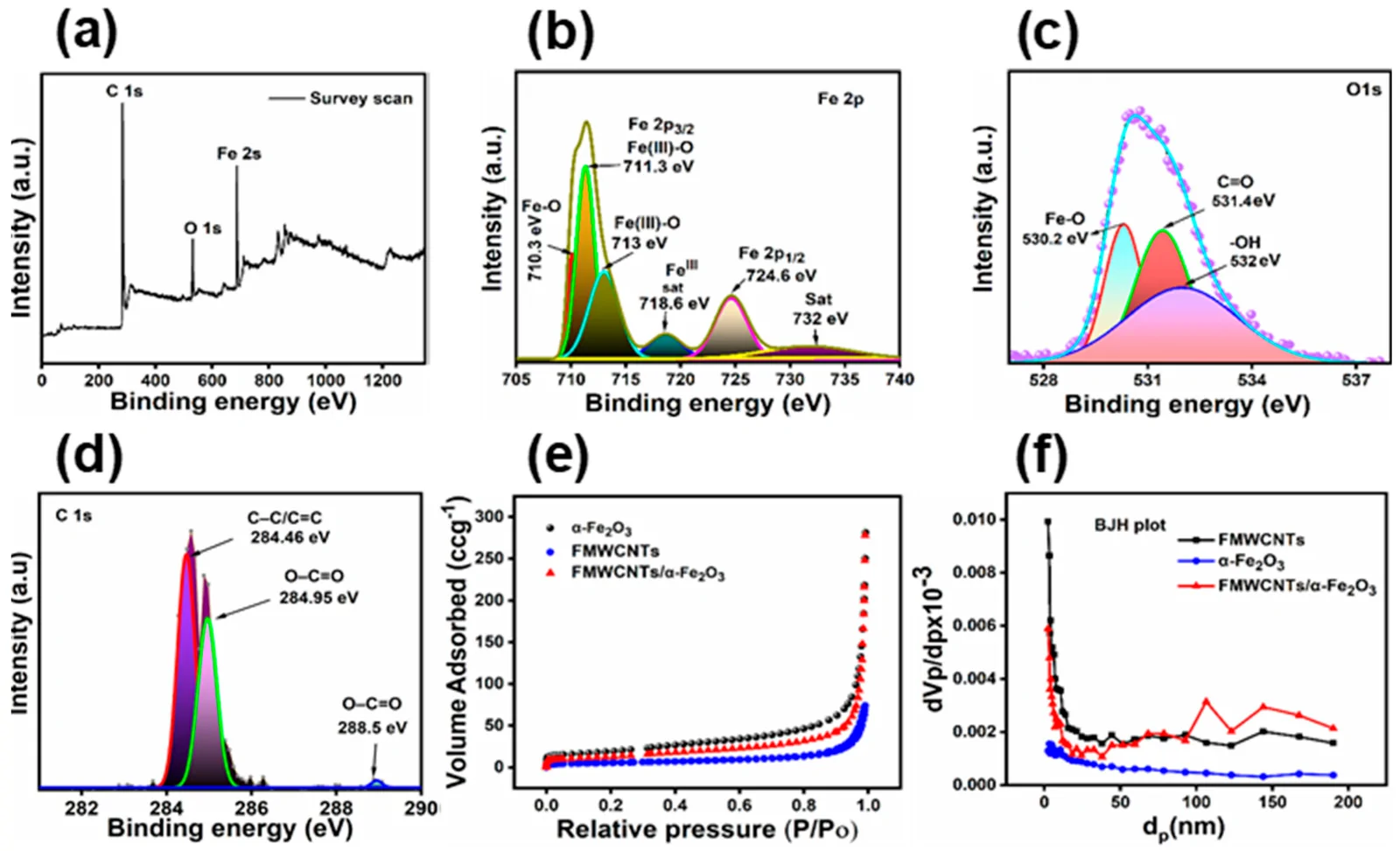
Chemical composition and surface analysis. (**a**) XPS survey scan spectrum of the FMWCNTs/α-Fe_2_O_3_ composite. High-resolution XPS spectra of (**b**) Fe 2p, (**c**) O 1s, and (**d**) C 1s. (**e**) N_2_ adsorption–desorption isotherms of the FMWCNTs, α-Fe_2_O_3_, and FMWCNTs/α-Fe_2_O_3_ electrodes. (**f**) BJH pore-size distribution curves for the as-prepared FMWCNTs, α-Fe_2_O_3_ (average pore diameter ~23 nm), and FMWCNTs/α-Fe_2_O_3_ (average pore diameter 37.7 nm) electrodes.

The elemental composition and chemical states of the prepared FMWCNTs/α-Fe_2_O_3_ composite were determined using XPS. The survey scan spectrum of the FMWCNTs/α-Fe_2_O_3_ composite shown in Figure 2a confirms the presence of Fe, O, and C. The detected carbon signal primarily originates from the FMWCNTs and carbon-containing surface functional groups introduced during functionalization. High-resolution XPS (HR-XPS) spectra provide detailed information on the oxidation states of Fe and O. Figure 2b shows the high-resolution Fe 2p XPS spectrum with multiple deconvoluted peaks confirming the presence of Fe^3+^ species. The Fe 2p_3/2_ spectrum exhibits components at 710.3, 711.3, and 713.0 eV, characteristic of Fe^3+^ species in the α-Fe_2_O_3_ phase. The Fe 2p_1/2_ peak at 724.6 eV, together with the characteristic shake-up satellite features at 718.6 and 732.0 eV, further corroborates the presence of Fe^3+^. Collectively, these XPS features confirm that Fe predominantly exists in the +3 oxidation state in the FMWCNTs/α-Fe_2_O_3_ composite. Additionally, the characteristic shake-up satellite peaks at 718.6 eV and 732.0 eV further validate the presence of Fe^3+^ oxidation states in the sample. These results clearly demonstrate that Fe predominantly exists in the Fe^3+^ state in the composite [50,51].

The O 1s spectrum (Figure 2c) was deconvoluted to differentiate the oxygen species in α-Fe_2_O_3_. The main peak at 530.2 eV is attributed to Fe–O in the α-Fe_2_O_3_ lattice. The peak at 531.4 eV is generally attributed to oxygen species associated with defects, such as oxygen vacancies, and to carbonyl (C=O) groups present in the functionalized MWCNTs. The peak at 532.0 eV is assigned to hydroxyl and possibly carbonate species. XPS alone is a semi-quantitative probe of oxygen vacancies. However, the relative enhancement of the 531.4 eV component indicates the presence of a significant amount of defect-related oxygen species in the composite. Defect-related oxygen species are generally reported to facilitate charge transfer and ion transport, which may contribute to the enhanced electrochemical performance of the composite. The interaction between the oxygen-containing functional groups of FMWCNTs and Fe–O species may enhance interfacial electronic coupling and improve charge-transfer kinetics [52,53].

The high-resolution C 1s spectrum of the composite (Figure 2d) shows a peak at 284.95 eV, corresponding to sp^3^-hybridized carbon. The peak at 284.46 eV is attributed to sp^2^-hybridized C–C/C=C bonds, consistent with the graphitic framework of FMWCNTs, whereas the component at 288.5 eV corresponds to O–C=O species associated with carboxyl groups introduced during acid functionalization. These spectral features indicate the preservation of the graphitic framework of FMWCNTs together with the presence of oxygen-containing functional groups. The oxygen-containing groups may provide favorable sites for interaction with α-Fe_2_O_3_ and may contribute to interfacial coupling and charge transfer within the composite. Such interfacial interactions could facilitate charge transport and contribute to the structural stability of the composite. Nevertheless, the XPS results provide supporting but indirect evidence of interfacial interactions, and a specific Fe–O–C chemical linkage cannot be conclusively established from XPS alone [53,54].

### 3.4. Surface Analysis

Mesoporosity and a high surface area provide a high density of electrochemically active sites, facilitating rapid ion diffusion and efficient charge storage, which are essential for achieving high specific capacitance and rate capability. The specific surface area and pore structure of FMWCNTs, α-Fe_2_O_3_, and FMWCNTs/α-Fe_2_O_3_ were characterized by Brunauer–Emmett–Teller (BET) and Barrett-Joyner-Halenda (BJH) analyses, as shown in Figure 2e,f. All samples exhibit type IV N_2_ adsorption–desorption isotherms with hysteresis loops in the relative pressure (*P*/*P0*) range of 0.4–1.0, indicative of mesoporous structures. The specific surface areas are 48.6 m^2^/g for FMWCNTs, 17.52 m^2^/g for α-Fe_2_O_3_, and 69.2 m^2^/g for the FMWCNTs/α-Fe_2_O_3_ composite. Notably, the FMWCNTs/α-Fe_2_O_3_ composite exhibits a significantly higher specific surface area than either pristine FMWCNTs or α-Fe_2_O_3_, indicating the formation of a hierarchical porous architecture. This enhancement is ascribed to the hierarchical organization created during the assembly of the composite. The FMWCNT network provides a conductive scaffold that inhibits the aggregation of α-Fe_2_O_3_ NSs. The insertion of α-Fe_2_O_3_ NSs between the CNT bundles prevents their re-aggregation. This mutual structural regulation results in more interparticle voids and secondary mesopores, thereby increasing the accessible surface area. This interpretation is further supported by the BJH pore-size distribution, in which the average pore diameter increases from ~23 nm for α-Fe_2_O_3_ to ~37.7 nm for the composite, indicating the development of interconnected mesoporous channels within the composite structure. This hierarchical porosity is beneficial for electrolyte penetration and reduces ion transport resistance during the charge–discharge process.

### 3.5. Three-Electrode Study

**Figure 3:**
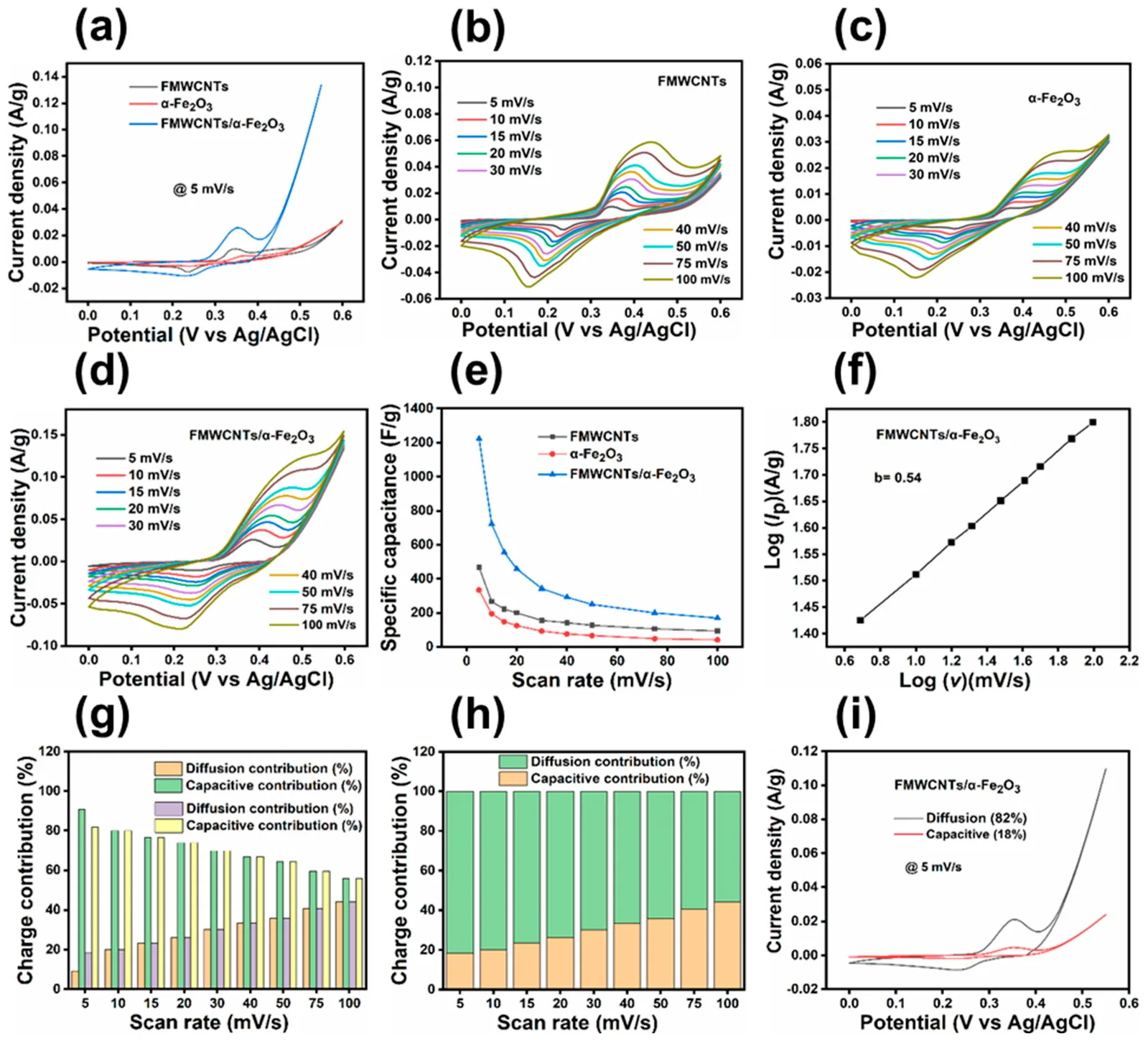
Electrochemical performance in a three-electrode system. (**a**) Comparative CV curves of all electrodes at a scan rate of 5 mV/s. CV curves of (**b**) FMWCNTs, (**c**) α-Fe_2_O_3_, and (**d**) FMWCNTs/α-Fe_2_O_3_ at scan rates ranging from 5 to 100 mV/s. (**e**) Plot of specific capacitance versus scan rate. (**f**) Determination of the *b*-value of the FMWCNTs/α-Fe_2_O_3_ electrode from the plot of log(*i*_p_) versus log(v). (**g**) Comparative charge contribution ratios of α-Fe_2_O_3_ and FMWCNTs/α-Fe_2_O_3_ electrodes at different scan rates. (**h**) Detailed charge contribution at various scan rates for FMWCNTs/α-Fe_2_O_3_. (**i**) Visual representation of capacitive and diffusive contributions for FMWCNTs/α-Fe_2_O_3_ at a scan rate of 5 mV/s.

The electrochemical performance of the as-prepared FMWCNT, α-Fe_2_O_3_, and FMWCNTs/α-Fe_2_O_3_ electrodes was characterized by CV, GCD, and EIS in a three-electrode configuration in 3 M KOH aqueous electrolyte. Figure 3a presents the CV curves of the FMWCNT, α-Fe_2_O_3_, and FMWCNTs/α-Fe_2_O_3_ electrodes at a scan rate of 5 mV/s. The α-Fe_2_O_3_ and FMWCNTs/α-Fe_2_O_3_ electrodes exhibit distinct redox peaks, confirming reversible Faradaic reactions. In comparison, the FMWCNT electrode displays a predominantly quasi-rectangular CV profile with weak redox humps, which can be associated with surface-controlled pseudocapacitive contributions from oxygen-containing functional groups in the FMWCNTs, in addition to electric double-layer capacitance (EDLC). However, because NF can also exhibit Faradaic activity in alkaline KOH electrolyte, the contribution of the current collector to the observed weak redox features was evaluated using a bare NF control CV at 100 mV/s (Figure S2c). The bare NF exhibits a distinct redox response; therefore, the weak humps observed for the FMWCNT electrode may contain a minor contribution from the NF substrate. The AC/NF control electrode was also evaluated by CV at a scan rate of 100 mV/s, together with the bare NF and FMWCNTs/α-Fe_2_O_3_ electrodes (Figure S2a). Compared with the FMWCNTs/α-Fe_2_O_3_ electrode, the AC/NF electrode exhibits a substantially smaller CV response and enclosed area, suggesting a comparatively lower charge-storage contribution from the activated carbon/binder-containing electrode components. Nevertheless, the predominantly quasi-rectangular profile of the FMWCNT electrode indicates that surface-controlled capacitive charge storage remains significant. During electrochemical cycling in the alkaline KOH electrolyte, OH^−^ ions actively participate in surface-driven Faradaic redox reactions. Although the XPS analysis (Section 3.3) confirms that the initial chemical state of iron in the composite is predominantly Fe^3+^, cycling in the highly alkaline medium may involve dynamic surface-state transformations. During the initial cycles or under cathodic polarization, a fraction of the surface Fe^3+^ species may undergo partial reduction to lower-valence states, such as Fe^2+^. These electrochemically generated species subsequently participate in reversible Faradaic reactions with OH^−^ ions during successive anodic and cathodic scans. This reversible Faradaic transition is formulated as follows:

2Fe(OH)_2_ + 2OH^−^ ↔ Fe_2_O_3_ + 3H_2_O + 2e^−^ (9)

The electrochemical mechanism of Fe_2_O_3_-based supercapacitors in alkaline electrolytes has been previously investigated, indicating the involvement of reversible Fe_2_O_3_/Fe(OH)_2_ redox transitions in charge storage [54]. This continuous Faradaic transition, combined with the highly accessible electroactive surface of the FMWCNT framework, accounts for the pronounced diffusion-controlled charge-storage behavior observed in the CV profiles.

CV curves of pristine FMWCNTs and α-Fe_2_O_3_ at various scan rates (5 to 100 mV/s, Figure 3b,c) exhibit non-rectangular shapes. At low scan rates, the characteristic redox peaks of α-Fe_2_O_3_ correspond to Fe^3+^/Fe^2+^ transitions, and those of the FMWCNTs originate from the surface oxygen functional groups. In contrast, at high scan rates, these peaks are less pronounced due to kinetic limitations because ion diffusion into the bulk becomes slower [55,56]. For the FMWCNTs/α-Fe_2_O_3_ composite (Figure 3d), enhanced redox currents and an enlarged CV area are observed, attributed to the synergistic effect between conductive FMWCNTs and redox-active α-Fe_2_O_3_. Defect-related oxygen species and interfacial electronic interactions may further contribute to improved charge-transfer kinetics and reversible Faradaic reactions. The specific capacitance values derived from the CV curves (Figure 3e) reach 1222.2 F/g for FMWCNTs/α-Fe_2_O_3_ at a scan rate of 5 mV/s, exceeding those of FMWCNTs (466.6 F/g) and α-Fe_2_O_3_ (333.3 F/g). The enhanced performance is attributed to abundant redox-active sites provided by Fe^3+^ transitions, enhanced conductivity, suppression of α-Fe_2_O_3_ agglomeration by the FMWCNT network, and hierarchical porosity enabling electrolyte penetration and efficient ion transport during cycling [50]. To further investigate the effect of FMWCNT content, the CV responses of FMWCNTs/α-Fe_2_O_3_ composites containing 10, 20, and 50 wt.% FMWCNTs were compared (Figure S3). The CV curves show an increasing current response and integrated CV area with increasing FMWCNT content, indicating enhanced electrochemical charge-storage behavior at higher FMWCNT loading. These results demonstrate that the FMWCNT content has a significant influence on the electrochemical response of the composite [57]. As the scan rate increases, the specific capacitance decreases due to insufficient time for electrolyte ions to penetrate the entire electrode structure. The *b*-value from the current-scan rate power-law analysis (Figure 3f) is approximately 0.54 for the FMWCNTs/α-Fe_2_O_3_ electrode, indicating predominantly diffusion-controlled, battery-type kinetics [58,59]. At 5 mV/s, the diffusion-controlled contribution accounts for approximately 82% of the total stored charge, whereas the capacitive contribution represents about 18% for the FMWCNTs/α-Fe_2_O_3_ electrode (Figure 3g,i). For comparison, the α-Fe_2_O_3_ electrode exhibits a substantially higher diffusion-controlled contribution of approximately 90.7%, with a corresponding capacitive contribution of 9.3% at 5 mV/s (Figure 3g), further confirming its predominantly battery-type charge-storage behavior. At higher scan rates, the capacitive contribution increases (Figure 3h), reflecting a shift from bulk diffusion-controlled storage toward a surface-limited process [60]. The above contributions were quantified by fitting the CV data to the Dunn relationship expressed in Equation (10).

(10) $$i_{p}=k_{1}V+k_{2}V^{\frac{1}{2}}$$

In this equation, k_1_ and k_2_ correspond to capacitive and diffusion-controlled contributions, respectively.

**Figure 4:**
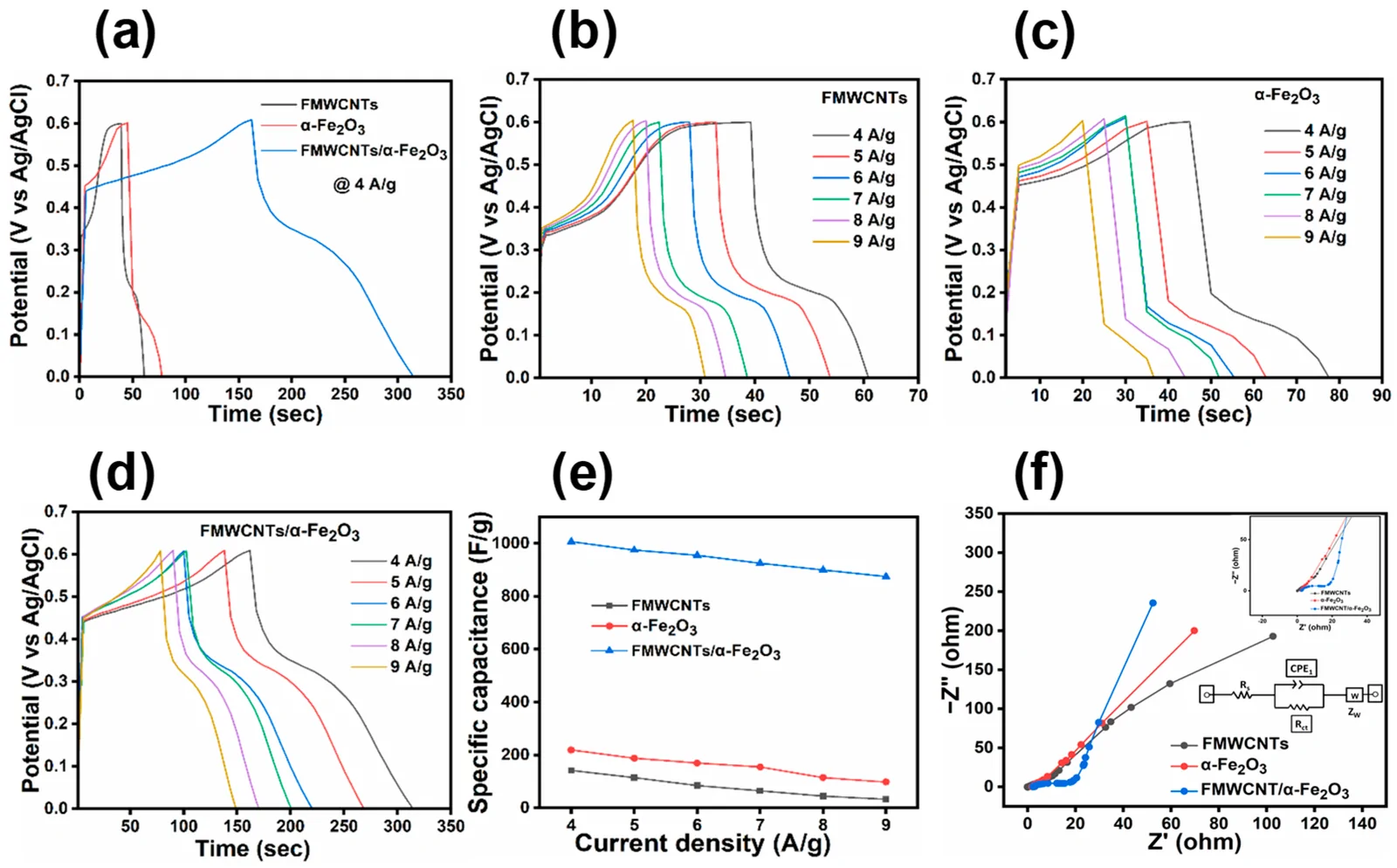
Galvanostatic charge–discharge and impedance analysis. (**a**) Comparative GCD curves of all electrode materials at a current density of 4 A/g. GCD profiles of (**b**) FMWCNTs, (**c**) α-Fe_2_O_3_, and (**d**) FMWCNTs/α-Fe_2_O_3_ at current densities ranging from 4 to 9 A/g. (**e**) Plot of specific capacitance versus current density. (**f**) Nyquist plots for FMWCNTs, α-Fe_2_O_3_, and FMWCNTs/α-Fe_2_O_3_ composite electrodes (Inset: Equivalent circuit model used for fitting).

The GCD technique was performed to evaluate the charge storage behavior and capacitive performance of the electrodes. The electrode performance of α-Fe_2_O_3_, FMWCNTs, and FMWCNTs/α-Fe_2_O_3_ was analyzed through a series of GCD tests at current densities from 4 to 9 A/g with 1 A/g increments, as shown in Figure 4. The GCD tests were performed at relatively high current densities (4 to 9 A/g) because the electrodes exhibit low ohmic resistance and rapid ion–electron transport. The non-linear GCD profiles in Figure 4a reflect distinct charge-storage kinetics across the samples. The pronounced curvature in α-Fe_2_O_3_ confirms diffusion-controlled battery-type Faradaic storage, whereas FMWCNTs display surface-dominated pseudocapacitive and EDLC behavior. The composite integrates both to deliver a synergistic hybrid charge-storage mechanism. The FMWCNTs/α-Fe_2_O_3_ electrodes exhibit distinctly nonlinear potential-time profiles reflecting reversible electrochemical events associated with ion uptake and release during the charge/discharge process. The smooth charge–discharge profiles indicate stable electrochemical behavior and reversible charge-storage processes.

The GCD curves of FMWCNTs at different current densities are shown in Figure 4b. In Figure 4c, the curvature of the charge/discharge profile of α-Fe_2_O_3_ confirms its diffusion-controlled, battery-type Faradaic nature, originating from reversible redox reactions. A small IR drop is observed at the beginning of the discharge process, which originates from the internal resistance of the electrode/electrolyte system [61,62]. The FMWCNTs/α-Fe_2_O_3_ composite exhibits a substantially longer discharge time than both pristine FMWCNTs and α-Fe_2_O_3_ NSs (Figure 4d), indicating a superior charge-storage capability arising from the synergistic combination of the conductive FMWCNTs and the redox-active α-Fe_2_O_3_ NSs. The specific capacitances of the individual FMWCNT and α-Fe_2_O_3_ electrodes were 234.6 and 288.0 F/g, respectively, at a current density of 4 A/g. In contrast, the composite electrode delivered a much higher specific capacitance of 1007.3 F/g at the same current density (Figure 4e). To further quantify the contribution of the electrode components, control electrodes of bare nickel foam (NF) and activated carbon deposited on nickel foam with binder (AC/NF) were also investigated under identical electrochemical conditions. At 4 A/g, the NF, AC/NF, and FMWCNTs/α-Fe_2_O_3_ electrodes exhibited specific capacitances of 24.6, 74.8, and 1007.3 F/g, respectively. The capacitances of the NF and AC/NF controls correspond to only 2.44% and 7.43%, respectively, of that of the FMWCNTs/α-Fe_2_O_3_ electrode. These values are presented as direct comparisons of the measured electrochemical responses rather than exact physical contributions of the individual electrode components. The substantially smaller responses of the NF and AC/NF controls support the dominant contribution of the FMWCNTs/α-Fe_2_O_3_ active composite to the measured electrochemical performance. To further justify the selection of the FMWCNT/α-Fe_2_O_3_ composition, GCD measurements were performed for composites containing 10, 20, and 50 wt.% FMWCNTs at 4 A/g (Figure S3b). The discharge time progressively increased with increasing FMWCNT content, indicating enhanced charge-storage capability. The corresponding specific capacitances were 127.2, 410.0, and 1007.3 F/g for the 10, 20, and 50 wt.% FMWCNTs/α-Fe_2_O_3_ electrodes, respectively (Figure S3c). The substantially higher capacitance obtained for the 50 wt.% FMWCNT composite indicates a composition-dependent enhancement that may be associated with improved interfacial charge transport and electrochemical utilization of the redox-active α-Fe_2_O_3_ phase. Among the investigated compositions, the 50 wt.% FMWCNT composite showed the highest specific capacitance. Therefore, the 50:50 composition was selected for subsequent investigations [63,64]. Furthermore, the FMWCNTs/α-Fe_2_O_3_ composite exhibits excellent stability, retaining approximately 85% of its initial capacitance after 10,000 GCD cycles at 4 A/g (Figure S3d). A comparative summary with previously reported studies is provided in Table S1 [65,66,67,68,69,70,71,72].

Key electrical properties of the electrodes, including electrical conductivity, ion mobility, and charge-transfer characteristics, were investigated using EIS. The results are presented in Figure 4f as a Nyquist plot (Z′ vs. −Z″) over the frequency range of 100 mHz to 100 kHz. The Nyquist plot highlights the electrochemical characteristics of the electrode, showing two distinctive features: a semicircular arc in the high-frequency region and a linear tail in the low-frequency region.

To provide a clearer view of the resistance values, an enlarged version and the corresponding equivalent circuit diagram used for fitting are shown in the inset of Figure 4f. The high-frequency intercept of the semicircle on the real axis corresponds to the solution/series resistance (*R*_s_), which comprises the contact resistance at the material substrate interface, the resistance of the substrate itself, and the ionic resistance of the electrolyte. The diameter of the semicircle represents the charge-transfer resistance (*R*_ct_, *R*_2_) associated with reversible electrochemical reactions. The markedly reduced *R*_ct_ reflects enhanced charge-transfer kinetics and improved OH^−^/K^+^ ion transport in the KOH electrolyte, attributed to the conductive FMWCNT network and the porous composite architecture of the FMWCNTs/α-Fe_2_O_3_ electrode. These features facilitate rapid electron transfer and efficient diffusion of electrolyte ions. The NS-decorated nanotube morphology is beneficial for improving electrolyte accessibility and reducing OH^−^ ion-diffusion pathways [61]. The *R*_s_ values are 4.2, 11.7, and 9.4 Ω for the FMWCNTs/α-Fe_2_O_3_, FMWCNT, and α-Fe_2_O_3_ electrodes, respectively. The relatively higher *R*_s_ of the FMWCNT electrode may be associated with its higher overall ohmic and interfacial resistance, which can arise from differences in electrode–substrate contact, electrode morphology, and the physicochemical characteristics of the functionalized FMWCNT network. Similarly, the *R*_ct_ values determined from the semicircle diameters were 0.39, 1.3, and 0.92 Ω for the FMWCNTs/α-Fe_2_O_3_, FMWCNT, and α-Fe_2_O_3_ electrodes, respectively. To account for non-ideal capacitive behavior stemming from surface heterogeneity and distributed charge transfer, constant phase elements (CPE1 and CPE2) were used in place of ideal capacitors. These results are attributed to the following key factors: (i) the interconnected FMWCNT network provides continuous electron-transport pathways, and the anchored α-Fe_2_O_3_ NSs shorten the ion-diffusion length, together facilitating rapid charge transport, (ii) the hierarchical surface morphology supplies abundant electroactive sites and maintains open porosity, supporting efficient electrode-electrolyte interactions, and (iii) the low interfacial resistance suppresses ohmic losses during cycling.

### 3.6. Electrochemical Performance of Ti_3_C_2_T_x_-MXene Electrode

**Figure 5:**
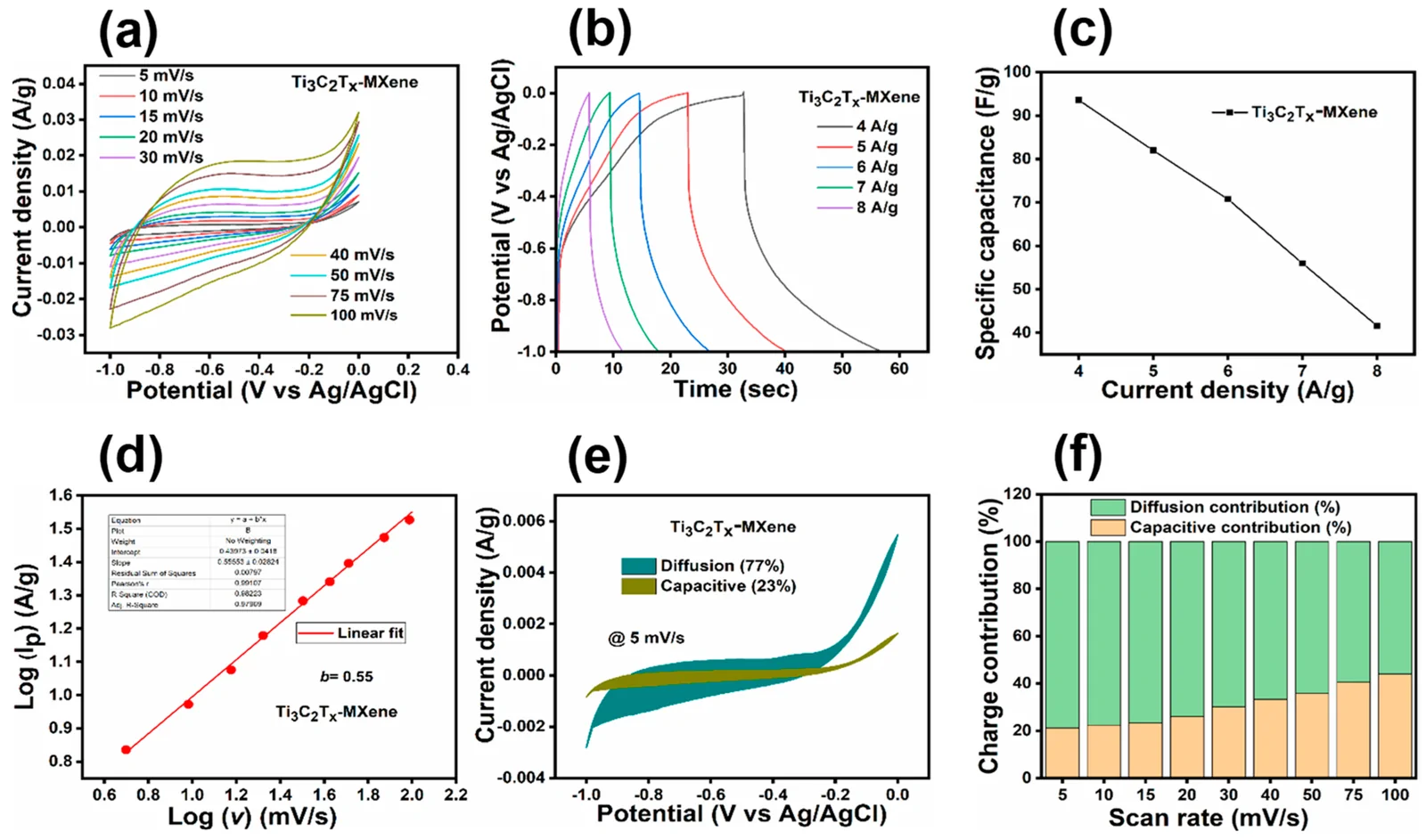
Electrochemical characterization of the Ti_3_C_2_T_x_-MXene anode. (**a**) CV curves of Ti_3_C_2_T_x_-MXene at various scan rates. (**b**) GCD curves of Ti_3_C_2_T_x_-MXene at different current densities. (**c**) Plot of specific capacitance versus current density. (**d**) Plot of log(ip) versus log(v) for the determination of the *b*-value. (**e**,**f**) Analysis of capacitive and diffusive contributions at different scan rates for Ti_3_C_2_T_x_-MXene.

The electrochemical performance of the Ti_3_C_2_T_x_-MXene electrode was assessed by CV, GCD, and kinetic analyses over a potential range of −1.0 to 0.0 V at scan rates of 5 to 100 mV/s. The CV profiles shown in Figure 5a are quasi-rectangular but slightly distorted, revealing fast surface redox reactions coupled with ion intercalation behavior, rather than a purely capacitive response. The kinetic analysis identifies the diffusion-controlled contribution as dominant at low scan rates, indicating charge storage governed predominantly by ion intercalation with a secondary surface-capacitive contribution [62,73]. The GCD curves at different current densities (3 to 5 A/g, Figure 5b) are quasi-symmetric, indicating good electrochemical reversibility and stable charge storage behavior. The specific capacitance derived from the GCD curves (Figure 5c) decreases with increasing current density, with a maximum value of 343.3 F/g at 3 A/g [22,74]. The relatively high specific capacitance of the Ti_3_C_2_T_x_ electrode may be associated with accessible electrochemically active sites, favorable ion transport within the layered MXene structure, and the influence of surface termination chemistry on accessibility and charge-storage behavior. Previous studies have demonstrated that the electrochemical performance of Ti_3_C_2_T_x_ in alkaline electrolytes is strongly influenced by its surface terminations and the interaction of electrolyte cations with the MXene layers. The relatively high capacitance may reflect differences in electrode structure, accessible electrochemical surface sites, surface termination chemistry, and ion transport behavior. However, the individual contribution of these factors cannot be quantitatively separated from the present measurements [75,76]. The Nyquist plot of the Ti_3_C_2_T_x_-MXene electrode is presented in Figure S4. The spectrum exhibits a relatively small high-frequency intercept followed by a steep, inclined low-frequency region, indicating low solution/series resistance and a predominantly capacitive/diffusion-related electrochemical response.

To investigate the charge storage mechanism, the *b*-value is determined from the plot of log(*i*_p_) versus log(v) (Figure 5d). The obtained value of 0.55, lying between 0.5 and 1.0 but close to 0.5, indicates a predominantly diffusion-limited process [74]. To quantify these charge contributions, the procedure proposed by Dunn was employed. At a low scan rate of 5 mV/s, a diffusion-controlled contribution of 77% is observed and the remaining 23% originates from the capacitive process (Figure 5e,f) [77]. These findings establish Ti_3_C_2_T_x_-MXene as an efficient negative electrode enabling fast and reversible charge/discharge in the assembled ASC.

### 3.7. Theoretical Insights into the Enhanced Electrochemical Behavior of FMWCNTs/α-Fe_2_O_3_ Hybrid Electrodes

**Figure 6:**
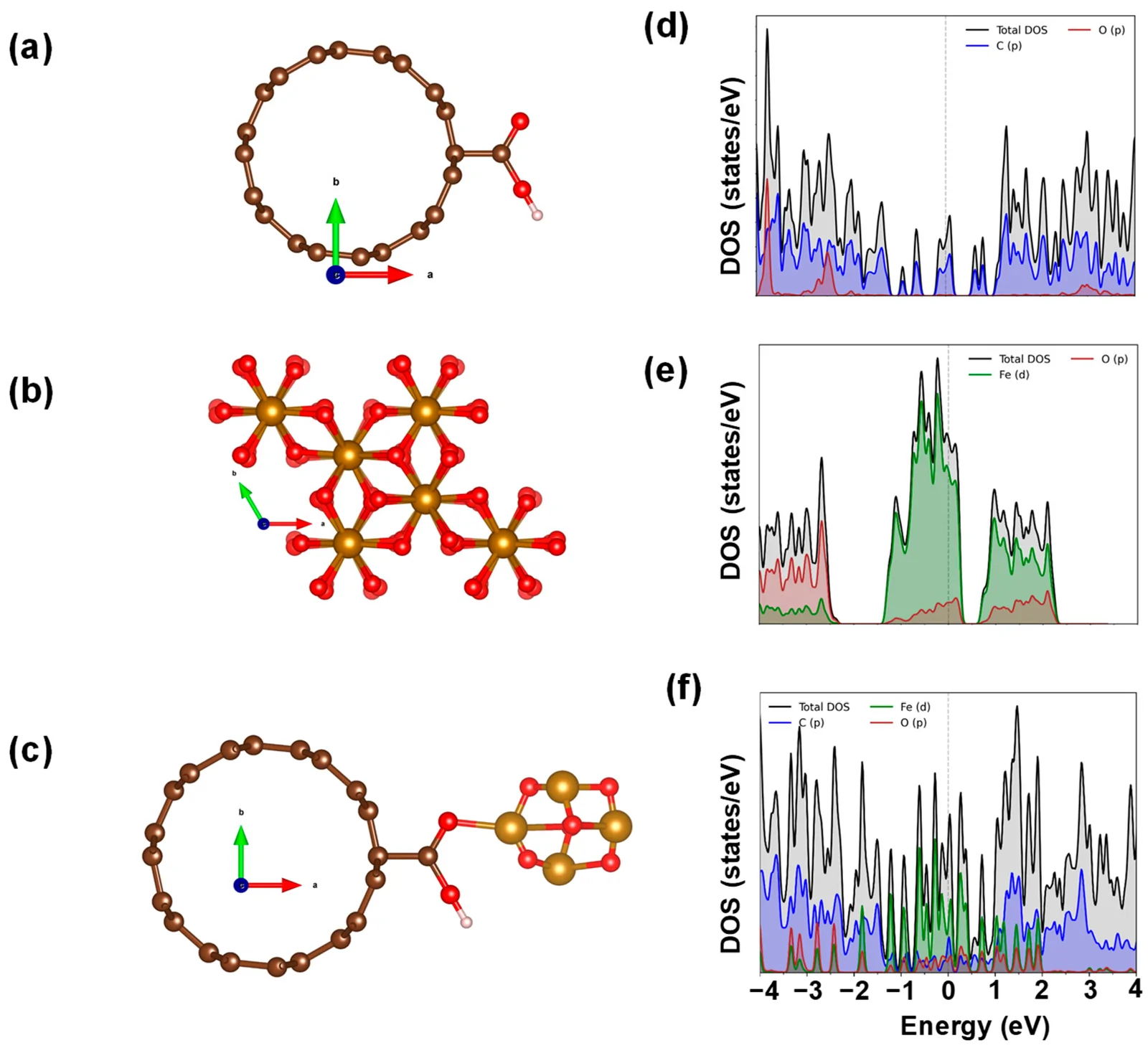
(**a**–**c**) Atomic structures of pristine FMWCNTs, bare α-Fe_2_O_3_, and the FMWCNTs/α-Fe_2_O_3_ HTs, respectively (brown, red, gold, and white spheres represent C, O, Fe, and H atoms; red, green, and blue arrows indicate the x-, y-, and z-axes, respectively). Density of states (DOS) plots of (**d**) FMWCNTs, (**e**) α-Fe_2_O_3_, and (**f**) the FMWCNTs/α-Fe_2_O_3_ HTs. The Fermi level is set to 0 eV.

To evaluate the intrinsic electronic properties underlying the charge-storage behavior of the FMWCNT, α-Fe_2_O_3_, and FMWCNTs/α-Fe_2_O_3_ electrodes, DFT calculations were performed on their atomic structures and electronic density of states (DOS), as depicted in Figure 6. The optimized atomic configurations of FMWCNTs, α-Fe_2_O_3_, and the FMWCNTs/α-Fe_2_O_3_ composite are presented in Figure 6a–c, respectively. The FMWCNT is represented by a carboxyl-functionalized nanotube (Figure 6a), the tubular carbon framework of which acts as a conductive scaffold with high electron mobility. In contrast, the α-Fe_2_O_3_ model (Figure 6b) consists of octahedrally coordinated Fe–O units, characteristic of the stable bonding environment of transition-metal oxides. After hybridization (Figure 6c), the α-Fe_2_O_3_ unit is anchored to the FMWCNT surface through the carboxyl group, suggesting a possible interfacial configuration involving Fe, O, and C atoms. The DOS analysis of the hybrid structure (Figure 6f) reveals electronic interactions among the C, O, and Fe states, which may facilitate electron transport and contribute to enhanced charge-storage performance. The electronic density of states (DOS) profiles of FMWCNTs, α-Fe_2_O_3_, and FMWCNTs/α-Fe_2_O_3_ are shown in Figure 6d–f. The continuous electronic states near the Fermi level (EF) in Figure 6d are predominantly contributed by C 2p orbitals, highlighting the conductive nature of FMWCNTs. In contrast, α-Fe_2_O_3_ (Figure 6e) exhibits predominantly Fe 3d and O 2p states, reflecting its distinct electronic structure and relatively lower intrinsic electrical conductivity compared with FMWCNTs. The overlap between the Fe 3d, O 2p, and C 2p orbitals suggests electronic interaction and possible orbital coupling at the FMWCNTs/α-Fe_2_O_3_ heterointerface. This electronic interaction may contribute to charge transport and improved electrochemical kinetics. Although the present DFT model employs an idealized, defect-free heterointerface to isolate the intrinsic orbital hybridization (Fe 3d, O 2p, and C 2p) and electronic coupling between the FMWCNTs and α-Fe_2_O_3_, the calculated electronic structure should be considered qualitative and may not fully represent the structural complexity of the experimentally synthesized composite. As indicated by the XPS analysis (Section 3.3), the oxygen-containing species and functional groups may contribute to interfacial interactions; however, their specific contribution to the electronic properties cannot be directly established from the present calculations. Therefore, the enhanced electrochemical performance of the FMWCNTs/α-Fe_2_O_3_ electrode is attributed to the synergistic effect of conductive FMWCNTs and redox-active α-Fe_2_O_3_, which collectively promote faster electron transport and improved reaction kinetics [78]. This interfacial electronic interaction is consistent with the observed diffusion-controlled Faradaic charge storage and the reduced charge-transfer resistance obtained from the EIS measurements. Overall, the DFT results provide qualitative support for possible electronic interactions at the FMWCNTs/α-Fe_2_O_3_ interface, while the specific nature of Fe–O–C bonding in the experimental composite cannot be conclusively established from the present calculations alone.

### 3.8. Two-Electrode Study

**Figure 7:**
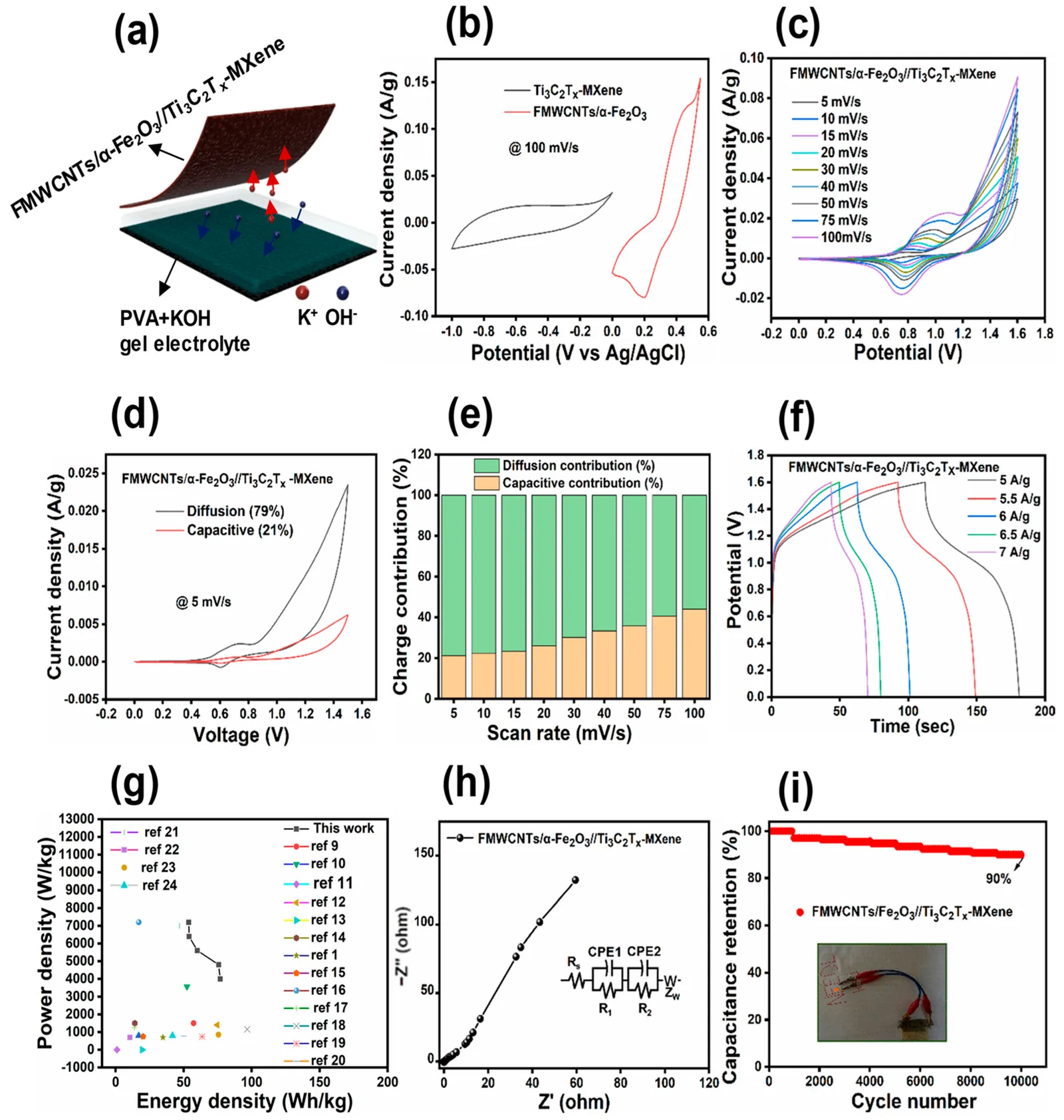
Performance of the asymmetric supercapacitor device. (**a**) Schematic illustration of the assembled ASC device. (**b**) CV curves of Ti_3_C_2_T_x_-MXene and FMWCNTs/α-Fe_2_O_3_ electrodes at 100 mV/s showing the operating potential windows. (**c**) CV curves of the optimized device at scan rates from 5 to 100 mV/s. (**d**,**e**) Capacitive and diffusive contribution analysis of the FMWCNTs/α-Fe_2_O_3_//Ti_3_C_2_T_x_-MXene ASC. (**f**) GCD curves at different current densities. (**g**) Ragone plot comparing the energy and power densities of the prepared device with other reported systems. (**h**) Nyquist plot of the ASC device (Inset: Equivalent circuit). (**i**) Long-term cycling stability test over 10,000 cycles with an inset showing an LED powered by the device.

The ASC device was fabricated using FMWCNTs/α-Fe_2_O_3_ as the cathode and Ti_3_C_2_T_x_-MXene as the anode to assess its practical energy storage capabilities. A schematic of the device design is shown in Figure 7a. The charge storage behavior primarily involves the movement and adsorption of OH^−^ ions at the cathode/electrolyte interface. The complementary operating windows of the two electrodes, 0 to 0.6 V for FMWCNTs/α-Fe_2_O_3_ and −1.0 to 0 V for Ti_3_C_2_T_x_-MXene (Figure 7b, at 100 mV/s), enable a broad device voltage window of 1.6 V, facilitating greater charge accumulation. To fully exploit the 1.6 V operational window and prevent premature overpolarization of either electrode, the active mass ratio of the cathode to the anode was optimized prior to device assembly based on the charge-balance principle (*q*_+_ = *q*_−_). Utilizing the single-electrode specific capacitances (1007.3 F/g for the cathode and 343.3 F/g for the anode) and their respective potential windows (*ΔV*_+_ = 0.6 *V*, *ΔV*_−_ = 1.0 V), the theoretical optimal mass ratio (*m*_+_/*m*_−_) was calculated to be approximately 0.57 (equivalent to 1:1.75). Consequently, the active mass loadings were adjusted to 2.0 mg for the cathode and 3.5 mg for the anode, and all device-level metrics were calculated based on the total active mass (5.5 mg).

The device exhibits pronounced redox peaks indicative of hybrid battery supercapacitor behavior and maintains its CV shape at high scan rates, demonstrating excellent reversibility and rate performance. CV analysis (Figure 7c) confirms the dual charge-storage nature of the device, in which the FMWCNTs/α-Fe_2_O_3_ cathode contributes Faradaic charge storage through reversible Fe^3+^/Fe^2+^ transitions, and the Ti_3_C_2_T_x_-MXene anode stores charge through diffusion-dominated ion intercalation/deintercalation with a minor capacitive contribution. The specific capacitance of the FMWCNTs/α-Fe_2_O_3_//Ti_3_C_2_T_x_-MXene device is 170.45 F/g at a scan rate of 5 mV/s (Figure S7b) [79,80,81]. The calculated *b*-value of 0.68 (Figure S7a) indicates mixed kinetics with a substantial diffusion-controlled component. According to the Dunn analysis, over 79% of the stored charge at lower scan rates originates from diffusion-controlled processes, emphasizing the dominant Faradaic contribution at both electrodes (Figure 7d,e).

To further investigate the charge-storage mechanism, post-cycling XPS analysis was performed on the FMWCNTs/α-Fe_2_O_3_ electrode. The Fe 2p spectrum after electrochemical cycling shows Fe^2+^- and Fe^3+^-related components, indicating the coexistence of different Fe oxidation states after cycling and providing additional oxidation-state-sensitive evidence consistent with the involvement of Fe redox chemistry in the charge-storage process. The O 1s spectrum retains the characteristic Fe–O contribution at approximately 530.3 eV, while the higher-binding-energy components are associated with oxygen-containing surface species. In addition, the C 1s spectrum exhibits a component near 292 eV, which can be attributed to –CF_2_– species originating from the PVDF binder. These post-cycling XPS results complement the b-value and Dunn analyses and provide additional evidence consistent with the contribution of Fe redox chemistry to the charge-storage process. Together with the high capacitance retention, these results suggest that the composite maintains its chemical integrity while retaining Fe redox activity during prolonged electrochemical cycling [82,83,84,85].

Typically, a dominant diffusion-controlled charge contribution indicates battery-type kinetics, which are often accompanied by considerable volume changes in the redox-active phase that may compromise long-term cycling stability. However, this trade-off is mitigated by the hierarchical architecture of the FMWCNTs/α-Fe_2_O_3_ HT. The highly interconnected carbon network shortens the ion-diffusion pathways within the nanoscale α-Fe_2_O_3_ domains, accelerating the diffusion-controlled charge storage. The GCD curves (Figure 7f) further confirm the performance of the ASC device, which delivered a specific capacitance of 220.2 F/g at 5 A/g (Figure S7c). The Ragone plot (Figure 7g and Figure S7d) illustrates a high energy density of 78.3 Wh/kg at a power density of 4072 W/kg (at 5 A/g), calculated from the measured discharge time at 5 A/g, surpassing many previously reported SC systems (Table S3) [86,87,88,89,90,91,92,93,94,95,96,97,98,99,100,101].

The Nyquist plot (Figure 7h) exhibits a small curved region in the high-frequency domain followed by a nearly linear inclined region at lower frequencies, indicating low interfacial resistance and diffusion-controlled ion transport within the porous electrode. The EIS data were fitted using the equivalent circuit shown in the inset of Figure 7h, consisting of a series resistance (*R*_s_) followed by two parallel *R*–CPE elements, (*R*1//CPE1) and (*R*_2_//CPE2), in series with a Warburg diffusion element (*W*). The fitted *R*_s_ value of 12.1 Ω indicates relatively low ohmic resistance, while the *R*_2_ (*R*_ct_) value of 0.58 Ω reflects low charge-transfer resistance and favorable interfacial electron-transfer kinetics. The Warburg coefficient of 45.2 Ω·s^−1/2^ indicates efficient ion diffusion within the porous electrode structure. The complete fitted equivalent-circuit parameters, including *R*_s_, *R*_ct_, CPE parameters, and the Warburg coefficient, are summarized in Table S2 in the Supplementary Materials. The two constant phase elements (CPE1 and CPE2) account for the non-ideal capacitive behavior associated with surface heterogeneity, distributed relaxation processes, and complex electrode-electrolyte interfacial charge-storage processes. In the HT electrodes, the adsorption/desorption of OH^−^ ions at the electrode–electrolyte interface proceeds rapidly and reversibly, together with efficient electron transfer to the current collector. The intertwined network of FMWCNTs and α-Fe_2_O_3_ NSs provides a high density of electroactive sites, significantly improving ion diffusion and accelerating the Faradaic process. In the cycling stability test (Figure 7i), the device retained 90% of its initial capacitance after 10,000 GCD cycles at 5 A/g, supporting its excellent durability. To further evaluate the structural and chemical changes after prolonged cycling, post-cycling SEM and XPS analyses were performed after 10,000 GCD cycles. The post-cycling SEM images show that the characteristic layered morphology of Ti_3_C_2_T_x_-MXene and the interconnected NSs on nanotubes of the FMWCNTs/α-Fe_2_O_3_ electrode remain observable after cycling. No obvious large-scale morphological collapse is observed in the examined SEM regions (Figure S5a,b). The Fe 2p XPS spectrum exhibits Fe^2+^- and Fe^3+^-related components, while the O 1s spectrum retains the characteristic Fe–O contribution at 530.3 eV, indicating that the Fe–O chemical environment remains detectable after cycling. The Ti 2p spectrum retains the Ti–C component at 460.7 eV together with Ti–O/Ti^4+^-related components at 458.4 and 464.2 eV, suggesting that the MXene-related Ti–C framework remains detectable, although some surface oxidation occurs during cycling. The C 1s spectrum also retains the C=C/C–C, C–O, and –CF_2_– components. Collectively, these post-cycling SEM and XPS observations indicate that the main morphological and chemical features remain detectable after prolonged cycling, providing supporting evidence for the observed 90% capacitance retention after 10,000 cycles [102,103]. The 90% capacitance retention over 10,000 cycles stems from a dual protective mechanism overcoming the intrinsic limitations of the active materials. In the assembled quasi-solid-state ASC, the PVA/KOH gel electrolyte plays a key role in establishing the quasi-solid-state architecture by immobilizing the KOH electrolyte within the PVA polymer matrix while maintaining ionic conductivity between the electrodes [104,105,106]. This gel configuration provides improved electrolyte retention and mechanical integrity, supporting stable operation of the assembled device. The PVDF binder primarily serves to maintain electrode integrity and good contact with the NF current collector during cycling. Thus, the PVA/KOH gel electrolyte is the primary polymeric component responsible for the quasi-solid-state configuration, while the PVDF binder provides complementary structural support to the electrodes. Structurally, the interwoven FMWCNT framework acts as an elastic buffer accommodating the volumetric strain of α-Fe_2_O_3_ and preventing severe pulverization during repetitive OH^−^ uptake. Chemically, pristine Ti_3_C_2_T_x_-MXene is known to be susceptible to anodic oxidation (forming inactive TiO_2_) in conventional aqueous alkaline solutions. However, the quasi-solid-state PVA–KOH gel electrolyte restricts the mobility of free water molecules and dissolved oxygen. This dense polymeric matrix acts as a protective barrier, suppressing the irreversible oxidative degradation of the MXene anode during prolonged cycling. The successful operation of a red LED at 1.6 V for 5 min (inset of Figure 7i) further confirms the practical applicability of the developed ASC.

## 4. Conclusions

Overall, the present work demonstrated the synergistic integration of FMWCNTs and α-Fe_2_O_3_ as an effective route to enhanced electrochemical performance in alkaline ASCs. The FMWCNTs and α-Fe_2_O_3_ were synergistically coupled to form a HT with a large surface area, enhanced porosity, and a favorable architecture facilitating ion transport and interfacial Faradaic reactions. These features enabled a high specific capacitance of 1007.3 F/g at a current density of 4 A/g. Moreover, the ASC device based on the Ti_3_C_2_T_x_-MXene anode exhibited a high energy density (78.3 Wh/kg) at a power density of 4072 W/kg, along with excellent rate performance and cycling stability, maintaining 90% of its capacitance after 10,000 cycles at a current density of 5 A/g. This study demonstrates the potential of the FMWCNTs/α-Fe_2_O_3_//Ti_3_C_2_T_x_-MXene system as a next-generation SC platform and provides a promising strategy for developing high-performance hybrid energy storage devices.

## Data Availability

The data supporting the findings of this study are available within the article and its Supplementary Materials.

## Supplementary Materials

The following supporting information can be downloaded at: https://www.mdpi.com/article/10.3390/polym18182262/s1, Figure S1: EDS spectrum and elemental quantification of the FMWCNTs/α-Fe_2_O_3_ composite; Figure S2: Comparison of the performance of bare nickel foam, AC/NF, and FMWCNTs/α-Fe_2_O_3_; Figure S3: Electrochemical performance of FMWCNTs/α-Fe_2_O_3_ electrodes with different FMWCNTs/α-Fe_2_O_3_ ratios; Figure S4: Nyquist plot obtained from electrochemical impedance spectroscopy (EIS) for the individual Ti_3_C_2_T_x_-MXene electrode. Figure S5: Post-cycling SEM images of the electrodes after 10,000 cycles; Figure S6: Post-cycling XPS analysis of the FMWCNTs/α-Fe_2_O_3_; Figure S7: Additional electrochemical performance metrics of the FMWCNTs/α-Fe_2_O_3_//Ti_3_C_2_T_x_-MXene ASC device; Table S1: Comparison of the electrochemical performance of FMWCNTs/α-Fe_2_O_3_ with previously reported single electrodes; Table S2: Fitted equivalent-circuit parameters obtained from the EIS spectrum of the assembled ASC; Table S3: Comparison of the electrochemical performance of the FMWCNTs/α-Fe_2_O_3_//Ti_3_C_2_T_x_-MXene ASC with previously reported ASC. The references cited in Tables S1 and S3 are included in the main reference list [65,66,67,68,69,70,71,72] and [86,87,88,89,90,91,92,93,94,95,96,97,98,99,100,101], respectively.
