# Supplementary material for: Interfacial Synergy in Hierarchical FMWCNTs/α-Fe2O3 and Ti3C2Tx-MXene Electrodes for High-Performance Asymmetric Supercapacitors
Source: Polymers (Basel). 2026 Sep 16;18(18):2262. doi: 10.3390/polym18182262 (PMC13611762; doi:10.3390/polym18182262)
Supplement: Supplementary file 1 [file polymers-18-02262-s001.zip › polymers-4549419-supplementary.pdf]

Supporting Information

Interfacial Synergy in Hierarchical FMWCNTs/α-Fe_2O_3 and $Ti_3C_2T_x$-MXene Electrodes for High-Performance Asymmetric Supercapacitors

Abdul Mutlib [1,†], Jihyo Lim [1,†], Inkyum Kim [2], Aqsa Ghazal [1], Hyunwoo Cho [3], Ahsan Javed [4] and Daewon Kim [1,2,*]

[1] Department of Semiconductor Engineering, Kyung Hee University, 1732 Deogyeong-daero, Giheung-gu, Yongin 17104, Republic of Korea; abdul.mutlib@khu.ac.kr (A.M.); jihyo.lim@khu.ac.kr (J.L.); aqsa.ghazal@khu.ac.kr (A.G.)
[2] Department of Electronic Engineering, Institute for Wearable Convergence Electronics, Kyung Hee University, 1732 Deogyeong-daero, Giheung-gu, Yongin 17104, Republic of Korea; inkyum.kim@khu.ac.kr
[3] Department of Electronics and Information Convergence Engineering, Kyung Hee University, 1732 Deogyeong-daero, Giheung-gu, Yongin 17104, Republic of Korea; hyunwoo.cho@khu.ac.kr
[4] Department of Physics, Syed Babar Ali School of Science and Engineering, Lahore University of Management Sciences (LUMS), Lahore 54792, Pakistan; ahsan.javed@lums.edu.pk
* Correspondence: daewon@khu.ac.kr
† These authors contributed equally to this work.

Table of Contents

1. EDS spectrum and elemental quantification of the FMWCNTs/α-Fe_2O_3 composite

2. Comparison of the performance of bare nickel foam, AC/NF, and FMWCNTs/α-Fe_2O_3

3. Electrochemical performance of FMWCNTs/α-Fe_2O_3 electrodes with different FMWCNTs/α-Fe_2O_3 ratios

4. Nyquist plot obtained from electrochemical impedance spectroscopy (EIS) for the individual $Ti_3C_2T_x$-MXene electrode.

5. Post-cycling SEM images of the electrodes after 10,000 cycles

6. Post-cycling XPS analysis of the FMWCNTs/α-Fe_2O_3

7. Additional electrochemical performance metrics of the FMWCNTs/α-Fe_2O_3//$Ti_3C_2T_X$-MXene ASC device

8. Comparison of the electrochemical performance of FMWCNTs/α-Fe_2O_3 with previously reported single electrodes

9. Fitted equivalent-circuit parameters obtained from the EIS spectrum of the assembled ASC device

10. Comparison of the electrochemical performance of the FMWCNTs/α-Fe_2O_3//$Ti_3C_2T_X$-MXene ASC with previously reported asymmetric supercapacitors

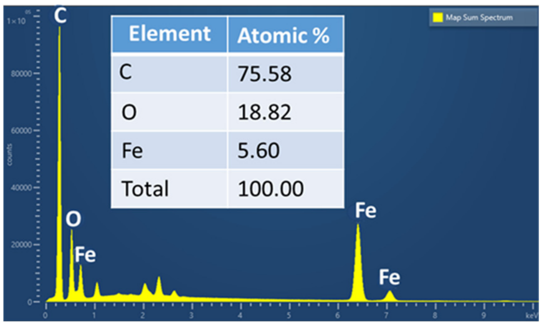

Figure S1. EDS spectrum and corresponding elemental quantification table for the FMWCNTs/α-Fe2O3 composite, confirming the presence of C, O, and Fe.

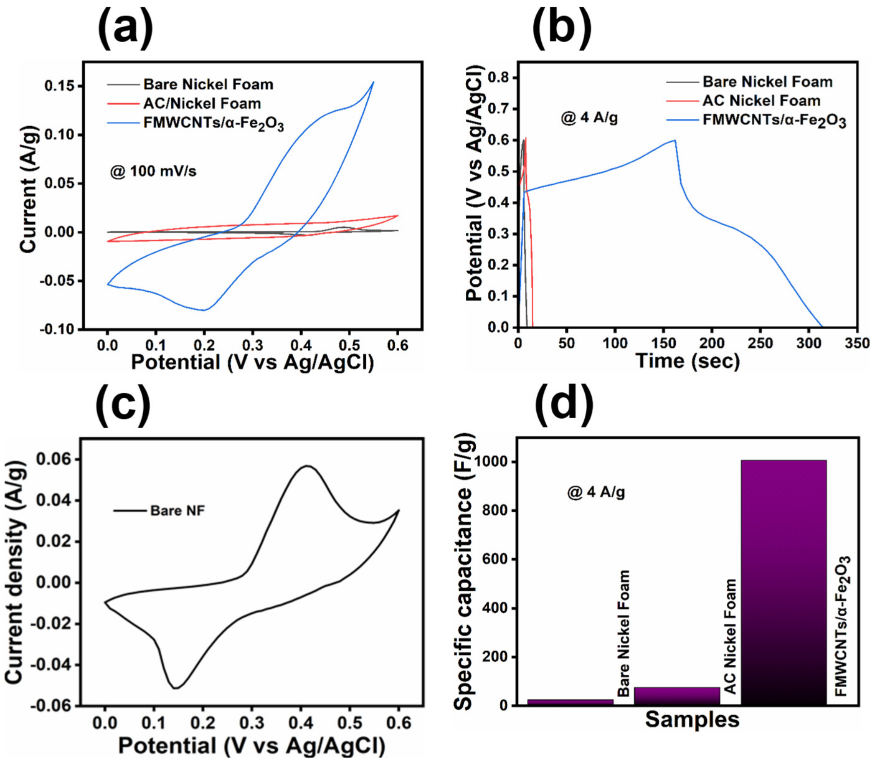

Figure S2. (a) CV curves of bare nickel foam (NF), activated carbon deposited on nickel foam with binder (AC/NF), and FMWCNTs/α-Fe_2O_3 electrodes at a scan rate of 100 mV/s in 3 M KOH; (b) GCD profiles of bare NF, AC/NF, and FMWCNTs/α-Fe_2O_3 electrodes at different current densities; (c) CV curves of bare NF at a scan rate of 100 mV/s; and (d) specific capacitance of bare NF, AC/NF, and FMWCNTs/α-Fe_2O_3 electrodes at a current density of 4 A/g.

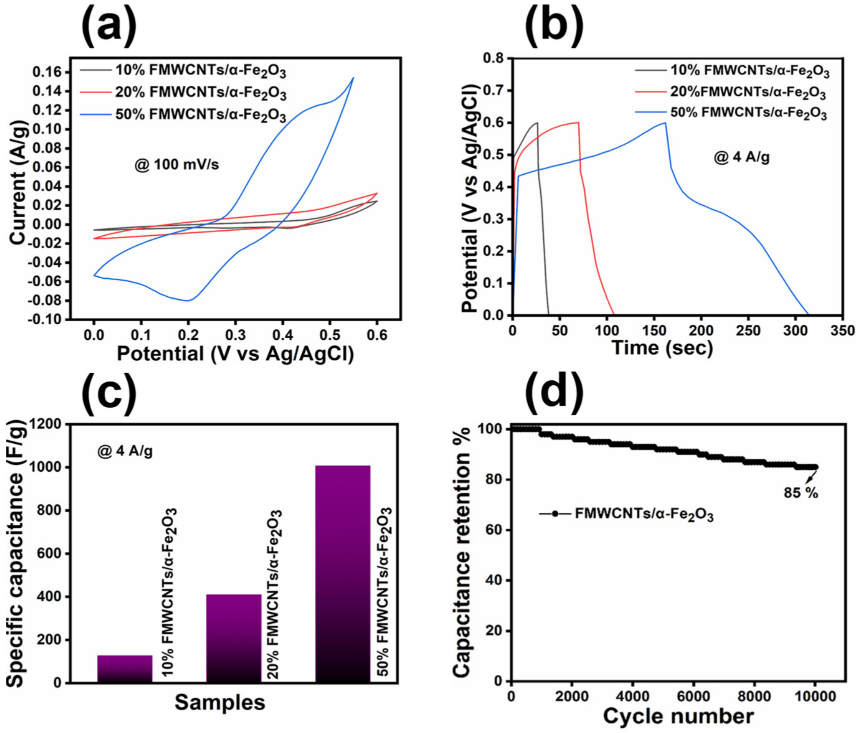

Figure S3. Electrochemical performance of FMWCNTs/α-Fe_2O_3 electrodes with different FMWCNTs/α-Fe_2O_3 ratios: (a) CV curves at 100 mV/s, (b) GCD profiles at 4 A/g, (c) specific capacitance comparison at 4 A/g, and (d) long-term cycling stability of the 50% FMWCNTs/α-Fe_2O_3 electrode over 10,000 GCD cycles at 4 A/g.

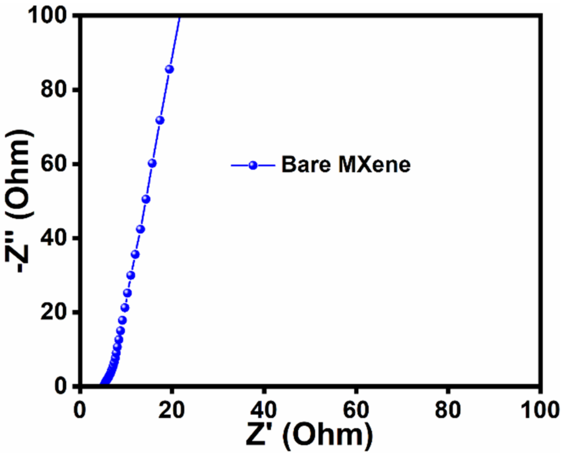

Figure S4. Nyquist plot obtained from electrochemical impedance spectroscopy (EIS) for the individual $Ti_3C_2T_x$-MXene electrode.

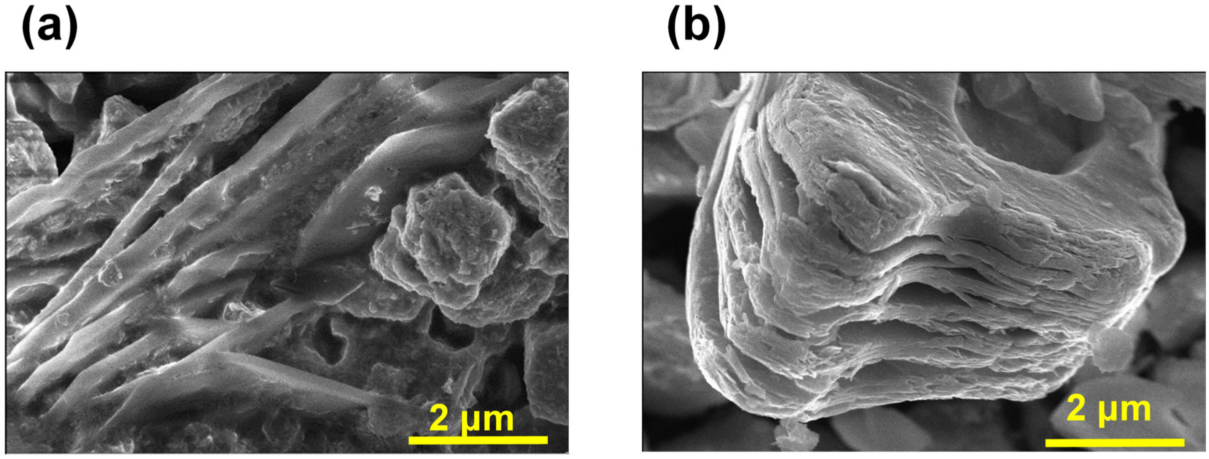

Figure S5. Post-cycling SEM images of the electrodes after 10,000 cycles: (a) SEM image of the FMWCNT/Fe_2O_3 cathode. (b) SEM image of the $Ti_3C_2T_x$ MXene anode.

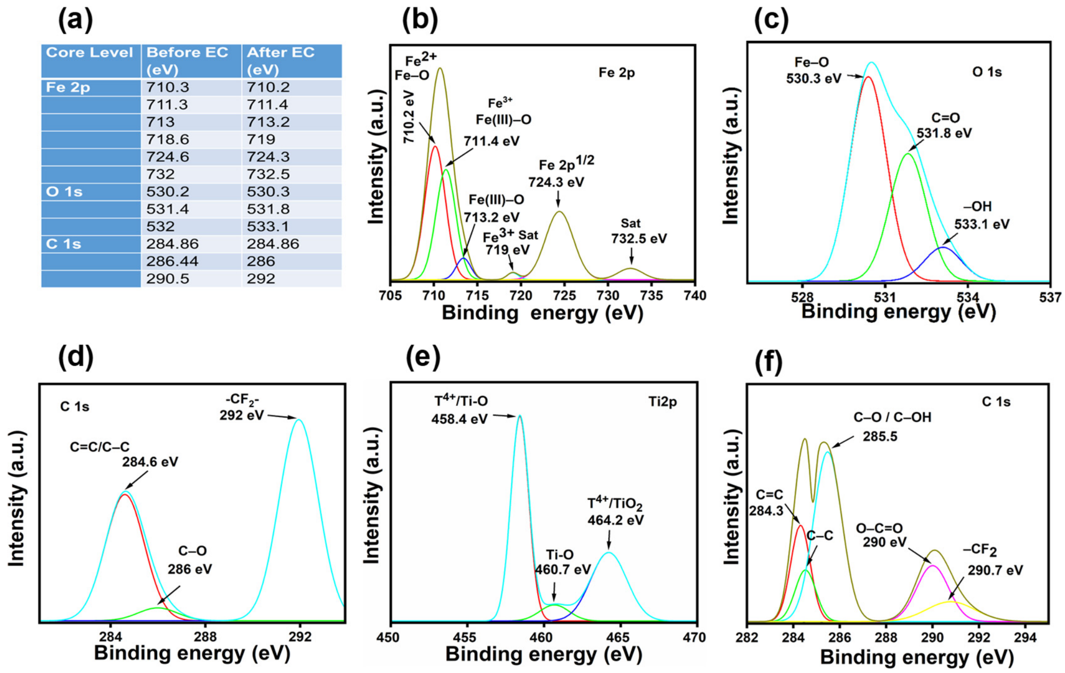

Core Level	Before EC (eV)	After EC (eV)
Fe 2p	710.3	710.2
	711.3	711.4
	713	713.2
	718.6	719
	724.6	724.3
	732	732.5
O 1s	530.2	530.3
	531.4	531.8
	532	533.1
C 1s	284.86	284.86
	286.44	286
	290.5	292

Figure S6. Post-cycling XPS analysis of the FMWCNTs/α-Fe_2O_3. (a) Table of core-level binding energies before and after electrochemical cycling. High-resolution XPS spectra of (b) Fe 2p, (c) O 1s, and (d) C 1s regions measured after electrochemical cycling, (e) Ti 2p, and (f) C 1s regions measured after cycling.

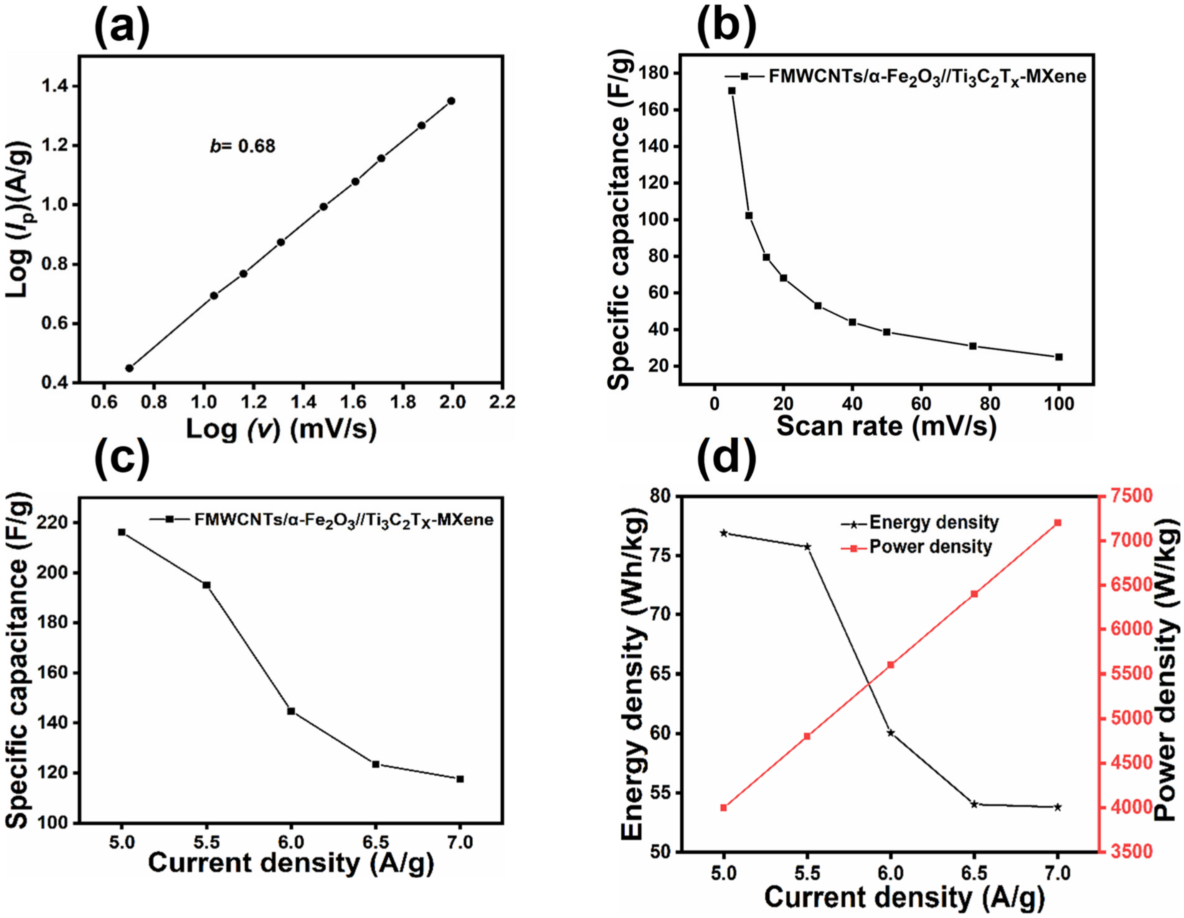

Figure S7. Additional electrochemical performance metrics of the ASC device. (a) Plot for determination of the *b*-value of the assembled device. (b) Specific capacitance of the device as a function of scan rate derived from CV analysis. (c) Specific capacitance of the device at various current densities. (d) Ragone plot showing power and energy densities at different current densities.

Table S1. Comparison of the electrochemical performance of the FMWCNTs/α-Fe_2O_3 with previously reported single electrodes.

No.	Material	Electrolyte	Specific capacitance (F/g)	Retention % After Cycles	Ref.
1	MOG/CNT-10	1 M Li_2SO_4	431.6	89.2% after 3,000 cycles	[1]
2	α-Fe_2O_3/rGO	1 M KOH	970	75% after 2,000 cycles	[2]
3	α-Fe_2O_3 HRs/rGO	2 M KOH	426.3	87.7% after 10,000 cycles	[3]
4	α-Fe_2O_3/NiO/rGO	6 M KOH	747	98% after 10,000 cycles	[4]
5	α-Fe_2O_3/MnO_2/rGO	6 M KOH	447	96% after 10,000 cycles	[5]
6	α-Fe_2O_3/rGO	2 M KOH	455	87.5% after 10,000 cycles	[6]
7	CC/CNT-$CuNiSe_2$	1 M KOH	957.06	N/A	[7]
8	α-Fe_2O_3@CeO_2	2 M Na_2SO_4	158	87.5% after 2,000 cycles	[8]
9	FMWCNTs/α-Fe_2O_3	3 M KOH	1007.3	85% after 10,000 cycles	This work

Table S2. Fitted equivalent-circuit parameters obtained from the EIS spectrum of the assembled FMWCNTs/α-Fe_2O_3//$Ti_3C_2T_x$-MXene (ASC).

**Variables**	**Values**
R_s (Ω)	12.1
R_1 (Ω)	4.5
R_{ct} (Ω)	0.58
CPE_1 ($S.s^n$)	0.00018
CPE_2 ($S.s^n$)	0.0036
Z_w ($\Omega \cdot s^{-1/2}$)	45.2

Table S3. Comparison of the electrochemical performance of the FMWCNTs/α-Fe_2O_3//$Ti_3C_2T_x$-MXene ASC with previously reported asymmetric supercapacitors.

No.	Material	Synthesis Method	Specific capacitance (F/g)	Energy Density (Wh/kg)	Power Density (W/kg)	Retention % after Cycles	Ref.
1	CNT/FPO//FPO/CNT	Chemical bath deposition	N/A	17	800	87% after 2,000 cycles	[9]
2	MWCNTs/$Co_3Fe_4(PO_4)_6$//$Co_3Fe_4(PO4)_6$ /MWCNTs	Chemical bath deposition	171	52.46	3563	98.9% after 5,000 cycles	[10]
3	$CoFe_2O_4$/CNTs//AC	One-pot hydrothermal	32.5	30.4	3986	85.6% after 1,000 cycles	[11]
4	GF–CNT@Fe_2O_3//GF–$CoMoO_4$	CVD (for carbon materials) and ALD (for metal oxide)	115.5	74.7	1400	95.4% after 5,000 cycles	[12]
5	MnO_2//10%La-Fe_2O_3@GO	Hydrothermal	46.9	19.5	900	87% after 5,000 cycles	[13]
6	NCP@CNT10@PPy6//AC ASC	Hydrothermal	127.9	34.8	700	75% after 8,000 cycles	[14]
7	MOG/CNT-10//AC QLHC	Sol–gel method	64.5	20.2	750	89.4% after 8,000 cycles	[1]
8	$BiFeO_3$ SSD	Sol–gel method	85	17.01	7200	88% after 5,000 cycles	[15]
9	ZnO-III (ZnO-III//AC)	Hydrothermal	199.99	13.88	1250	87.85% after 10,000 cycles	[16]
10	ZnO/3 wt.%MWCNTs//AC	Ultrasonication	84	N/A	N/A	N/A	[17]
11	CNTs@MnO_2 cathode//Nb_2CT_x	N/A	192.8	96.7	1156.3	88.7% after 10,000 cycles	[18]
12	NiCoLDH@α-Fe_2O_3(2:1)//CAC	Solvothermal	203.6	63.6	749	85% after 10,000 cycles	[19]
13	$NiFe_2O_4$/NiCo LDH@rGO//AC	N/A	155	50	780	93.02% after 3,000 cycles	[20]
14	α-Fe_2O_3/rGO//AC	N/A	150	47	7000	94% after 8,000 cycles	[21]
15	CoO/rGO//RI-2	Microwave synthesis	N/A	10.5	695	83% after 10,000 cycles	[22]
16	Fe_2O_3@NCS8//Fe_2O_3@C	Hydrothermal	188.23	75.6	850.5	90.5% after 10,000 cycles	[23]
17	CuCoSe@NiS/NF//AC	Hydrothermal	117.4	41.8	800	100% after 5,000 cycles	[24]
18	FMWCNTs/α-Fe_2O_3//$Ti_3C_2T_x$-MXene	Hydrothermal	220.2	78.3	4072	90% after 10,000 cycles	This work

References

[1] Y. Zhang, Q. Xie, R. Shao, J. Ding, J. Liu, W. Xu, Y. Wang, Green synthesis of MOF/CNT gels via in-situ physical mixing strategy toward quasi-solid-state Li-ion hybrid capacitor, J. Energy Storage 86 (2024) 111156. https://doi.org/10.1016/j.est.2024.111156.

[2] L. Chen, D. Liu, P. Yang, Preparation of α-Fe_2O_3/rGO composites toward supercapacitor applications, RSC Adv. 9 (2019) 12793–12800. https://doi.org/10.1039/c9ra01928f.

[3] G. Zhou, G. Liang, W. Xiao, L. Tian, Y. Zhang, R. Hu, Y. Wang, Porous α-Fe_2O_3 Hollow Rods/Reduced Graphene Oxide Composites Templated by MoO_3 Nanobelts for High-Performance Supercapacitor Applications, Molecules 29 (2024). https://doi.org/10.3390/molecules29061262.

[4] G. Mummoorthi, S. Shajahan, M. Abu Haija, U. Mahalingam, R. Rajendran, Synthesis and Characterization of Ternary α-Fe_2O_3/NiO/rGO Composite for High-Performance Supercapacitors, ACS Omega 7 (2022) 27390–27399. https://doi.org/10.1021/acsomega.2c02418.

[5] M. Geerthana, S. Prabhu, R. Ramesh, Hierarchical α-Fe_2O_3/MnO_2/rGO ternary composites as an electrode material for high performance supercapacitors application, J. Energy Storage 47 (2022) 103529. https://doi.org/10.1016/j.est.2021.103529.

[6] Y. Dong, L. Xing, F. Hu, A. Umar, X. Wu, α-Fe_2O_3/rGO nanospindles as electrode materials for supercapacitors with long cycle life, Mater. Res. Bull. 107 (2018) 391–396. https://doi.org/10.1016/j.materresbull.2018.07.038.

[7] S. Jana, S. Karingula, A. Sajeevan, V.V.N. Phani Kumar, Y.G. Kotagiri, In situ synthesis of bimetallic chalcogenides with highly conductive carbon nanotubes for efficient symmetric hybrid supercapacitors, Nanoscale 17 (2025) 13283–13297. https://doi.org/10.1039/d5nr00340g.

[8] M. Mazloum-Ardakani, F. Sabaghian, M. Yavari, A. Ebady, N. Sahraie, Enhance the performance of iron oxide nanoparticles in supercapacitor applications through internal contact of α-Fe_2O_3@CeO_2 core-shell, J. Alloys Compd. 819 (2020) 1–8. https://doi.org/10.1016/j.jallcom.2019.152949.

[9] T.B. Deshmukh, A.C. Mendhe, C. Padwal, D. Dubal, D.Y. Kim, B.R. Sankapal, Unlocking the synergetic potential of cobalt iron phosphate and multiwalled carbon nanotube composites towards supercapacitor application, J. Mater. Chem. A 12 (2024) 9896–9908. https://doi.org/10.1039/d4ta00247d.

[10] L. Yue, S. Zhang, H. Zhao, Y. Feng, M. Wang, L. An, X. Zhang, J. Mi, One-pot synthesis $CoFe_2O_4$/CNTs composite for asymmetric supercapacitor electrode, Solid State Ionics 329 (2019) 15–24. https://doi.org/10.1016/j.ssi.2018.11.006.

[11] R. Li, W. Li, Y. Li, S. Zhu, X. Ba, Y. Zhou, N-Doped Carbon Coated Bimetallic $CoFe_2O_4$ Nanomembranes as an Enhanced Negative Electrode for Asymmetric Supercapacitors, ChemNanoMat 10 (2024) 1–7. https://doi.org/10.1002/cnma.202400176.

[12] C. Guan, J. Liu, Y. Wang, L. Mao, Z. Fan, Z. Shen, H. Zhang, J. Wang, Iron oxide-decorated carbon for supercapacitor anodes with ultrahigh energy density and outstanding cycling stability, ACS Nano 9 (2015) 5198–5207. https://doi.org/10.1021/acsnano.5b00582.

[13] S.C. Pathan, N.S. Shaikh, S.S. Mali, J. V. Patil, P.K. Katkar, N.S. Padalkar, S. Praserthdam, C.K. Hong, P. Kanjanaboos, J.S. Shaikh, Unlocking the potential of La-doped iron oxide @graphene oxide and ionic liquid-based asymmetric supercapacitor, J. Energy Storage 55 (2022) 105642. https://doi.org/10.1016/j.est.2022.105642.

[14] X. Shi, L. Sun, X. Li, L. Wu, J. Qian, J. Wang, Y. Lin, S. Su, C. Sun, Y. Zhang, Y. Zhang, High-performance flexible supercapacitor enabled by Polypyrrole-coated NiCoP@CNT electrode for wearable devices, J. Colloid Interface Sci. 606 (2022) 135–147. https://doi.org/10.1016/j.jcis.2021.08.016.

[15] A.K. Nayak, T. Gopalakrishnan, Phase- and Crystal Structure-Controlled Synthesis of Bi_2O_3, Fe_2O_3, and BiFeO3Nanomaterials for Energy Storage Devices, ACS Appl. Nano Mater. 5 (2022) 14663–14676. https://doi.org/10.1021/acsanm.2c03026.

[16] A. Hussain, A. Tufail, A. Shakoor, M. Mehak, M.S. Akhtar, S.M. Ramay, S. Sarwar, S. Atiq, synergistically tailored ionic conduction and transport in ZnO/CNTs based electrodes with enhanced electrochemical efficiency in supercapacitors, Electrochim. Acta 536 (2025) 146736. https://doi.org/10.1016/j.electacta.2025.146736.

[17] N. Eswaramoorthy, S. Rajendran, B. Arjun Kumar, S. Nallusamy, M. Rengasamy, Y. Selvaraj, S. Sangaraju, T. Krishnan, G. Kumaresan, K. Rajaram, Influence of ZnO/MWCNTs based hybrid electrodes for boosting the performance of photovoltaic and supercapacitor devices, Mater. Chem. Phys. 316 (2024) 129049. https://doi.org/10.1016/j.matchemphys.2024.129049.

[18] Z. Zou, H. Chen, Q. Li, Y. Wei, X. Zhang, X. Zou, X. Mei, J. Tao, Excellent Zinc-Ion Hybrid Capacitor Based on the Nb_2CT_x Anode and CNTs@MnO_2 Nanocomposite Cathode, ACS Appl. Nano Mater. 7 (2024) 20645–20652. https://doi.org/10.1021/acsanm.4c03621.

[19] H. Rangaraju, P. Matheswaran, M. Govindasamy, B. Parasuraman, K. Shanmugaraj, P. Thangavelu, Hierarchical nanoengineered NiCo LDH@α-Fe_2O_3 heterostructures for optimised electrochemical capacitive performance, Mater. Chem. Phys. 340 (2025) 130822. https://doi.org/10.1016/j.matchemphys.2025.130822.

[20] D. Chu, F. Li, X. Song, H. Ma, L. Tan, H. Pang, X. Wang, D. Guo, B. Xiao, a novel dual-tasking hollow cube $NiFe_2O_4$-NiCo-LDH@rGO hierarchical material for high preformance supercapacitor and glucose sensor, J. Colloid Interface Sci. 568 (2020) 130–138. https://doi.org/10.1016/j.jcis.2020.02.012.

[21] S. Abbas, T.H. Bokhari, M. Abbas, Z. Abbas, A. Khalid, S. Javed, A. Zafar, N. Ahmad, S. Karim, A. Javed, T. Zhu, A. Nisar, M. Ahmad, High performance α-Fe_2O_3/rGO based prototype asymmetric coin cell supercapacitor in hybrid electrolyte, Electrochim. Acta 517 (2025) 145761. https://doi.org/10.1016/j.electacta.2025.145761.

[22] C.K. Maity, A. Sood, R. Singhmar, J.H. Choi, A. Milton, S.M. Choi, S. Sahoo, S.S. Han, Reduced graphene oxide-supported microwave synthesis of Hemin-derived Fe_2O_3-based composites for advanced supercapacitor devices, J. Alloys Compd. 1014 (2025) 178734. https://doi.org/10.1016/j.jallcom.2025.178734.

[23] S. Che, Z. Huang, S. Gao, M. Guo, Application of Fe_2O_3-based composites as both cathode and anode: Increasing matching degree to achieve enhanced supercapacitor energy density, J. Energy Storage 134 (2025) 118225. https://doi.org/10.1016/j.est.2025.118225.

[24] W. Wu, Y. Yan, X. Wang, C. Wei, N. Yang, T. Xu, X. Li, Interface construction of CuCoSe@NiS based on an ultrathin nanosheet for high-performance supercapacitors, J. Mater. Chem. A 12 (2024) 13818–13829. https://doi.org/10.1039/d4ta01430h.
